# Amyloid beta protein-induced zinc sequestration leads to synaptic loss via dysregulation of the ProSAP2/Shank3 scaffold

**DOI:** 10.1186/1750-1326-6-65

**Published:** 2011-09-22

**Authors:** Andreas M Grabrucker, Michael J Schmeisser, Patrick T Udvardi, Magali Arons, Michael Schoen, Nathaniel S Woodling, Katrin I Andreasson, Patrick R Hof, Joseph D Buxbaum, Craig C Garner, Tobias M Boeckers

**Affiliations:** 1 Institute for Anatomy and Cell Biology, Ulm University, Albert Einstein Allee 11, Ulm, 89081, Germany; 2 Department of Psychiatry and Behavioral Sciences, Stanford School of Medicine, Stanford University, 1201 Welch Road, Stanford, CA 94305-5485, USA; 3 Department of Neurology and Neurological Sciences, Stanford School of Medicine, Stanford University, 300 Pasteur Drive, Stanford, CA 94305 USA; 4 Fishberg Department of Neuroscience, Mount Sinai School of Medicine, One Gustave L. Levy Place, New York, NY 10029, USA; 5 Friedman Brain Institute, Mount Sinai School of Medicine, One Gustave L. Levy Place, New York, NY 10029, USA; 6 Department of Psychiatry, Mount Sinai School of Medicine, One Gustave L. Levy Place, New York, NY 10029, USA; 7 Department of Genetics and Genomic Science, Mount Sinai School of Medicine, One Gustave L. Levy Place, New York, NY 10029, USA

**Keywords:** PSD, Alzheimer's disease, ProSAP2, Shank3, Shank1, Amyloid, Oligomers, Zn^2+^, Hippocampus, synapse

## Abstract

**Background:**

Memory deficits in Alzheimer's disease (AD) manifest together with the loss of synapses caused by the disruption of the postsynaptic density (PSD), a network of scaffold proteins located in dendritic spines. However, the underlying molecular mechanisms remain elusive. Since it was shown that ProSAP2/Shank3 scaffold assembly within the PSD is Zn^2+^-dependent and that the amyloid beta protein (Aβ) is able to bind Zn^2+^, we hypothesize that sequestration of Zn^2+ ^ions by Aβ contributes to ProSAP/Shank platform malformation.

**Results:**

To test this hypothesis, we designed multiple *in vitro *and *in vivo *assays demonstrating ProSAP/Shank dysregulation in rat hippocampal cultures following Aβ oligomer accumulation. These changes were independent from alterations on ProSAP/Shank transcriptional level. However, application of soluble Aβ prevented association of Zn^2+ ^ions with ProSAP2/Shank3 in a cell-based assay and decreased the concentration of Zn^2+ ^clusters within dendrites. Zn^2+ ^supplementation or saturation of Aβ with Zn^2+ ^ions prior to cell treatment was able to counter the effects induced by Aβ on synapse density and ProSAP2/Shank3 levels at the PSD. Interestingly, intracellular Zn^2+ ^levels in APP-PS1 mice and human AD hippocampus are reduced along with a reduction in synapse density and synaptic ProSAP2/Shank3 and Shank1 protein levels.

**Conclusions:**

We conclude that sequestration of Zn^2+ ^ions by Aβ significantly contributes to changes in ProSAP2/Shank3 platforms. These changes in turn lead to less consolidated (mature) synapses reflected by a decrease in Shank1 protein levels at the PSD and decreased synapse density in hippocampal neurons.

## Background

The loss of synapses is closely associated with the cognitive impairment seen in patients with Alzheimer's disease (AD) [1-3]. Recent findings suggest that this loss is mediated by increasing levels of amyloid beta protein (Aβ), a product of amyloid precursor protein (APP) metabolism [4-6], although the mechanisms through which Aβ accumulation finally leads to synaptic degeneration are not fully understood. However, Pham et al. have recently shown that Aβ oligomers progressively accumulate in brains of AD patients as well as in APP transgenic mice together with a reduction in the levels of synaptic scaffold proteins such as Shank1 and ProSAP2/Shank3 [7].

Proteins of the ProSAP/Shank (Synamon, CortBP, Spank, SSTRIP) family play a crucial role in proper synapse function [8] and have been linked to autism, schizophrenia and AD [7-13]. Treatment of rat frontocortical neurons with soluble Aβ_1-40 _resulted in a significant thinning of the PSD and in decreased synaptic levels of Shank1 [13] and other ProSAP/Shank platform-associated PSD proteins such as PSD-95 [14], Homer [13] and GKAP/SAPAP [15]. Although the precise mechanism of ProSAP/Shank scaffold protein dysregulation still remains unclear, an emerging model is that alterations in those proteins could interfere with cognitive function and behavior by impairing excitatory glutamatergic synapses.

ProSAP/Shank platforms are organized through Zn^2+^-ions [16-18] and ProSAP/Shank protein levels depend on the local Zn^2+ ^concentration and influx [17]. Zn^2+ ^is found in PSDs and in synaptic vesicles at glutamatergic synapses throughout the neocortex and hippocampus and is released during synaptic activity [19]. Intriguingly, high concentrations of Zn^2+ ^are also observed in neuritic plaques and cerebrovascular amyloid deposits from both AD patients and AD-prone transgenic mice [20-22]. Aβ is a metal-binding protein with high affinity for copper and zinc [23,24] and Zn^2+ ^ions promote Aβ oligomerization [25].

In our study, we show that soluble oligomers of Aβ_1-40 _and Aβ_1-42 _induce changes in ProSAP/Shank protein levels at the synapse. These changes are not caused by a reduced ProSAP/Shank gene expression, but reflect an altered localization of ProSAP/Shank family members. Aβ seems to efficiently compete with Zn^2+ ^loading of ProSAP2/Shank3 finally leading to a decrease in dendritic Zn^2+ ^signals. The decline in synapse density and ProSAP2/Shank3 levels can be rescued by supplementation with Zn^2+^-ions or saturation of Aβ with Zn^2+^. Furthermore, in APP-PS1 mice and human AD brain sections, Zn^2+ ^sequestration in senile plaques is accompanied by a decrease in intracellular Zn^2+ ^concentration along with a decrease in synapse density and synaptic ProSAP2/Shank3 and Shank1 protein levels.

Thus, our results lead to a model illustrating that Aβ pathology is at least in part caused by trapping synaptic Zn^2+ ^in Aβ complexes, preventing Zn^2+ ^from reaching its postsynaptic targets like ProSAP/Shank proteins, ultimately leading to a dysregulation of the postsynaptic scaffold and subsequent loss of synapses which might in turn lead to the observed cognitive deficits in AD.

## Results

### Soluble Aβ oligomers induce changes in synapse density, maturation state and synaptic ProSAP2/Shank3 and Shank1 protein levels in primary hippocampal neurons

**Figure 1:**
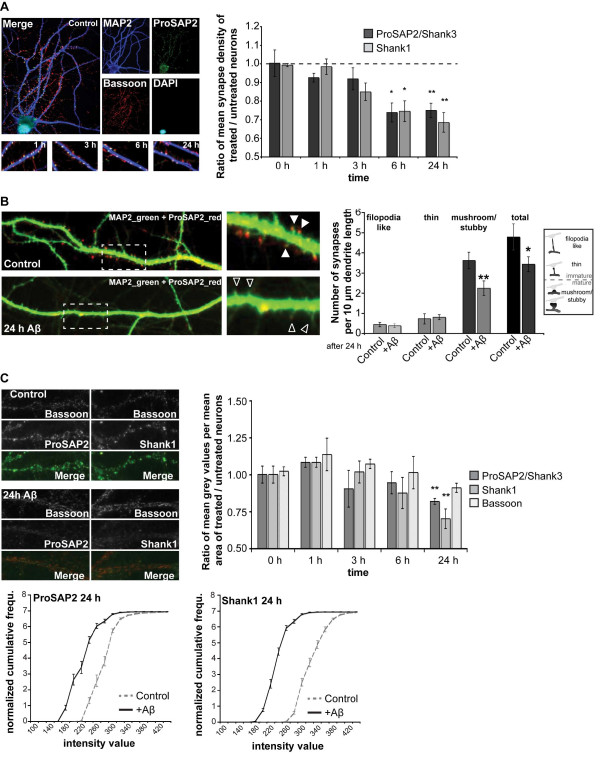
**Soluble Aβ oligomers reduce synapse density and dysregulate ProSAP/Shank family members in hippocampal cell culture**. A) Changes in synapse density along the dendrites of hippocampal neurons, cultured for 15 DIV, treated with 1 μM Aβ_1-40 _and fixed after 0, 1, 3, 6, and 24 h. Synapses along MAP2 positive dendrites were identified with antibodies against Bassoon as presynaptic marker and ProSAP2/Shank3 (left panel) or Shank1. Synapse density was calculated measuring the number of synapses per unit dendrite length of ten cells of three independent experiments for every time-point and condition (right panel). B) Spine maturation state after 24 h Aβ_1-40 _treatment was assessed by quantifying spine morphology (using ProSAP2/Shank3 immunoreactivity) along MAP2 positive dendrites (left panels). Spines were classified as "filopodia like", "thin" (immature) and "mushroom and stubby" (mature). The overall fraction of filopodia like and thin synapses is higher after 24 h Aβ treatment compared to control conditions (24 h treatment with DMSO). C) Aβ treatment causes a progressive synaptic loss of ProSAP2/Shank3 and Shank1. Cultured hippocampal neurons were immunostained with antibodies against Bassoon and ProSAP2/Shank3 or Shank1 (upper left panel) and the ratio of mean grey values per mean signal area between treated and untreated neurons were measured after 1, 3, 6 or 24 h treatment with Aβ_1-40 _(upper right panel). Cumulative histograms illustrate that the puncta intensity values are shifted across the entire populations of ProSAP2/Shank3 and Shank1 puncta (bottom panels). Data derive from 3 independent experiments at each time-point and condition representing approx. 2,500 signals per experiment.

Based on recent data showing that Aβ induces the disruption of the Homer1b and Shank1 scaffold [13], we investigated if soluble Aβ oligomers are sufficient to induce changes in ProSAP/Shank family members. We applied 1 μM Aβ_1-40 _or Aβ_1-42 _to rat primary hippocampal cell culture neurons (DIV15-17) and fixed them after 1, 3, 6 and 24 h, respectively. Immunohistochemistry was performed using anti-ProSAP2/Shank3 and anti-Shank1 antibodies co-stained with an anti-Bassoon antibody as a presynaptic marker. Synapse density was calculated by measuring the number of synapses (Bassoon and ProSAP/Shank positive sites) per unit dendrite length. The mean synapse density was significantly decreased after 6-24 h exposure to Aβ_1-40_, leading to a 30% reduction in synapse density after 24 h (Figure 1A and Aβ_1-42_, Additional file 1A).

To assess the maturation state of synapses, we characterized the morphology of dendritic spines in Aβ-treated cultures (Figure 1B). The results show that the proportion of "filopodia like" and "thin" spines, representing immature synapses with respect to the total synapse number, increased after 24 h Aβ treatment compared to control conditions (Figure 1B). This shift towards immature spines was accompanied by a decrease of mature ("mushroom and stubby") spines.

ProSAP/Shank family members are recruited to synapses in a sequential and development-dependent manner [17] beginning with ProSAP1/Shank2 that becomes concentrated at the sites where PSDs are thought to form [26], followed by ProSAP2/Shank3 protein. Finally, with sufficient amount of ProSAP1/Shank2 and ProSAP2/Shank3 present at the synapse, the clustering of Shank1 leads to maturation of the synaptic contacts and to spines with a mushroom-like appearance [27]. Hence, a shift towards immature spines should also influence the levels of Shank1 at synapses and we therefore measured the mean grey value and mean area of ProSAP2/Shank3 and Shank1 signals opposite to Bassoon signals (Figure 1C). In hippocampal neurons, ProSAP2/Shank3 and Shank1 proteins were significantly downregulated at the synapse after 24 h treatment with Aβ_1-40 _(18% ± 2% and 30 ± 7%, respectively; Aβ_1-42_, Additional file 1B) along with a downregulation of Homer1 and PSD-95 (Additional file 1C). The protein levels of Bassoon were not significantly affected (Figure 1C). A similar decrease was observed in cortical neurons, however here, a downregulation occurred as early as 1 h after treatment as reported previously [13] (Additional file 1D). The observed changes were caused by a decrease of protein levels at the synapse since the mean signal area was unaffected after Aβ treatment (Additional file 1E). Cumulative histograms illustrate that the puncta intensity values are shifted across the entire populations of ProSAP2/Shank3 and Shank1 puncta, revealing that mature synapses were affected by the treatment similarly to immature synapses (Additional file 1F). We thus conclude that exposure of neurons to Aβ causes the loss of synapses and that decreased ProSAP2/Shank3 and Shank1 levels following Aβ application, lead to altered maturation states of excitatory synapses.

### Aβ_1-40 _oligomer-induced changes in ProSAP/Shank protein levels are not mediated via transcriptional regulation

**Figure 2:**
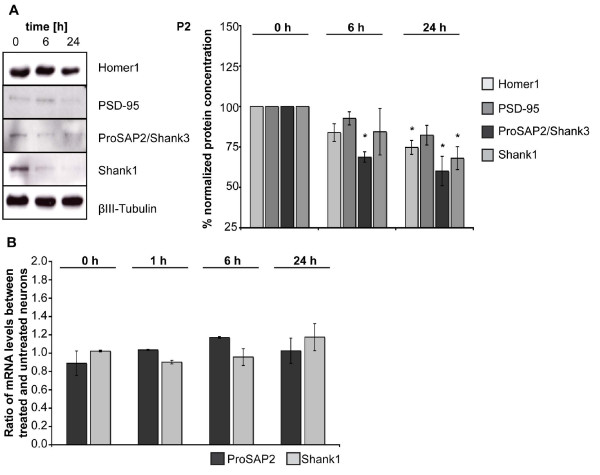
**Changes in synaptic ProSAP/Shank levels after Aβ treatment are not linked to transcriptional regulation**. Western blots of P2 membrane fractions from hippocampal neurons cultured for 15 DIV and then treated for 6 and 24 h with Aβ_1-40_. A) Compared to untreated cells at time-point 0, a significant decrease in the amount of ProSAP2/Shank3 and Shank1 could be detected after 6-24 h of Aβ treatment (right panel). Homer1 and PSD-95 levels also show a decrease of protein levels after 24 h. Lysates from 3 independent experiments were quantified via Western Blot analysis by measuring the integrated density. The values were normalized against β-III Tubulin and 0 h was set to 100%. B) Quantitative RT-PCR was performed for ProSAP2/Shank3 and Shank1 after Aβ_1-40 _treatment. mRNA was isolated from hippocampal neurons (DIV15) at time-point 0 and after 1, 6, and 24 h. The mean ratio between treated and untreated control neurons from three independent experiments is shown. No significant changes in transcription levels can be seen.

The changes in synaptic ProSAP/Shank levels after exposure to Aβ_1-40 _(1 μM) *in vitro *could further be confirmed by Western Blotting of P2 membrane fractions from hippocampal neurons at 15 DIV after Aβ-treatment for 6 and 24 h. Compared to untreated cells (time-point 0), significantly lower levels of ProSAP2/Shank3 (6 and 24 h) and Shank1 (24 h) within the P2 fraction of lysates could be detected after 24 h of Aβ_1-40 _treatment - similar to the effect observed by grey value measurement of immunohistochemical ProSAP2/Shank3 and Shank1 signals at the synapse (Figure 2A, for comparison see Figure 1C). After 24 h of treatment, Homer1 also showed a significant decrease in protein levels and PSD-95 a clear trend towards downregulation (Figure 2A, β-III Tubulin was used as control).

To assess if the observed changes in ProSAP/Shank protein levels at synapses were due to changes in gene expression levels, we performed quantitative RT-PCR (Figure 2B). Hippocampal neurons (DIV15) were treated with Aβ_1-40 _and mRNA was extracted after 1, 6, and 24 h. The results showed no significant differences in gene expression levels compared to controls indicating that the observed changes are due to a structural alteration of the PSD scaffold leading to a shift of ProSAP2/Shank3 from a PSD bound state to a soluble pool. Indeed, the ratio between ProSAP2/Shank3 within the S2 soluble (Additional file 2A) and P2 membrane (Figure 2A) fraction set to 1 at time-point 0 rises to 1.59 at 6 h and 1.69 at 24 h after treatment with Aβ. This is underlined by data showing that the reduction of ProSAP2/Shank3 and Shank1 at the synapse is independent of both, proteasomal degradation and protein synthesis, since treatment with the proteasome inhibitor MG132 or protein synthesis inhibitor cycloheximide (CHX) did not prevent Aβ_1-40_-induced changes in synaptic signal intensities of ProSAP2/Shank3 and Shank1. However, MK801, an NMDAR antagonist, significantly decreased the amount of Aβ_1-40_-induced changes in Shank1 levels as shown before [13] (Additional file 2B).

### Zinc sequestration by Aβ influences ProSAP2/Shank3 Zn^2+ ^loading and leads to lower intracellular Zn^2+ ^levels in hippocampal neurons

**Figure 3:**
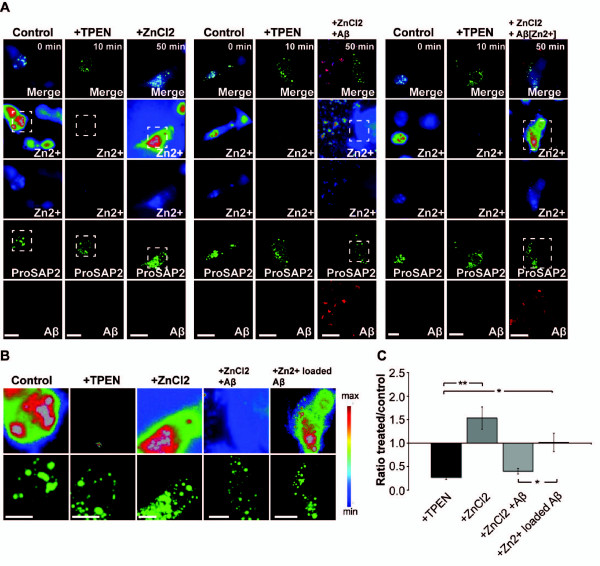
**Application of soluble Aβ oligomers decreases Zn^2+ ^loading of ProSAP2/Shank3**. Cos7 cells grown in 5 μM Zn^2+^-supplemented medium were transfected with GFP-ProSAP2/Shank3. The intracellular Zn^2+ ^level, visualized by Zinquin ethyl ester, and subcellular distribution were compared to GFP-ProSAP2/Shank3. A) In control cells, GFP-ProSAP2/Shank3 colocalizes with Zn^2+ ^(t = 0 min) (left panel). After application of TPEN, Zn^2+^-ions were removed from ProSAP2/Shank3 clusters (t = 10 min). Supplementation with 10 μM ZnCl_2 _restores and increases the initial Zn^2+^-association with GFP-ProSAP2/Shank3 clusters (left panel t = 50 min). Twenty min application of 10 μM Aβ_1-40_(red fluorescence) followed by supplementation with 10 μM ZnCl_2 _for 20 min only leads to a minor increase in Zn^2+ ^loading of ProSAP2/Shank3 (middle panel t = 50 min). Application of 10 μM Aβ_1-40_(red fluorescence) preloaded with 10 μM ZnCl_2 _followed by supplementation with 10 μM ZnCl_2 _leads to a significantly higher increase in ProSAP2/Shank3 Zn^2+ ^loading (right panel t = 50 min) (scale bar = 50 μm). B) Magnification of Zn^2+ ^signals colocalizing with ProSAP2/Shank3 cluster under the conditions described in A) (scale bar = 25 μm). C) Quantification of Zn^2+ ^fluorescence, visualized with Zinquin, colocalizing with ProSAP2/Shank3 clusters. The ratio of mean grey values between control cells (t = 0 min) and treated cells is shown.

Since ProSAP2/Shank3 protein levels at the PSD are sensitive to the local Zn^2+ ^concentration [17] and Aβ has a Zn^2+^-binding site and might thus be able to sequester Zn^2+ ^ions, we investigated if Aβ is indeed able to sequester extracellular Zn^2+ ^ions affecting the Zn^2+ ^loading of ProSAP2/Shank3. To that end, we transfected Cos7 cells growing in 5 μM Zn^2+^-supplemented medium with GFP-ProSAP2/Shank3 and depleted Zn^2+^-ions using TPEN (Figure 3A). After Zn^2+ ^depletion, Zn^2+ ^ions were introduced back into the medium via ZnCl_2 _with and without additional Aβ treatment. Furthermore, as a control, Aβ was preloaded with Zn^2+ ^ions and then added to the medium followed by ZnCl_2 _application. For these experiments, we took advantage of a dye (Zinquin) that fluoresces when it binds Zn^2+ ^[28] to measure the local Zn^2+ ^concentration (correlating with Zinquin signal intensity) colocalizing with GFP-ProSAP2/Shank3 clusters. The results show that in control cells, GFP-ProSAP2/Shank3 colocalizes with Zn^2+ ^(Figure 3A, t = 0 min, B). After 10 min application of the Zn^2+ ^chelator TPEN, Zn^2+^-ions were efficiently removed from ProSAP2/Shank3 clusters (Figure 3A, t = 10 min, B,C). Supplementation with 10 μM ZnCl_2 _restored and increased the initial Zn^2+ ^association of ProSAP2/Shank3 (Figure 3A left panel t = 50 min, B,C). However, 20 min application of 10 μM Aβ_1-40 _(red fluorescence) followed by supplementation of the medium with 10 μM ZnCl_2 _for 20 min only resulted in a minor increase in Zn^2+ ^loading of ProSAP2/Shank3 (Figure 3A, middle panel t = 50 min, B,C). In contrast, preloading of 10 μM Aβ_1-40 _(red fluorescence) with 10 μM ZnCl_2 _followed by supplementation of the medium with 10 μM ZnCl_2_, led to a significantly higher increase in ProSAP2/Shank3 Zn^2+ ^loading (Figure 3A, right panel, t = 50 min, B,C). Thus, Aβ influences Zn^2+ ^loading of ProSAP2/Shank3 by sequestering extracellular Zn^2+^-ions. Because Zn^2+^-ions pass through the extracellular space into the postsynaptic compartment after activity-dependent vesicle release, it could well be that Aβ oligomers accumulating in the synaptic cleft interfere with this process.

**Figure 4:**
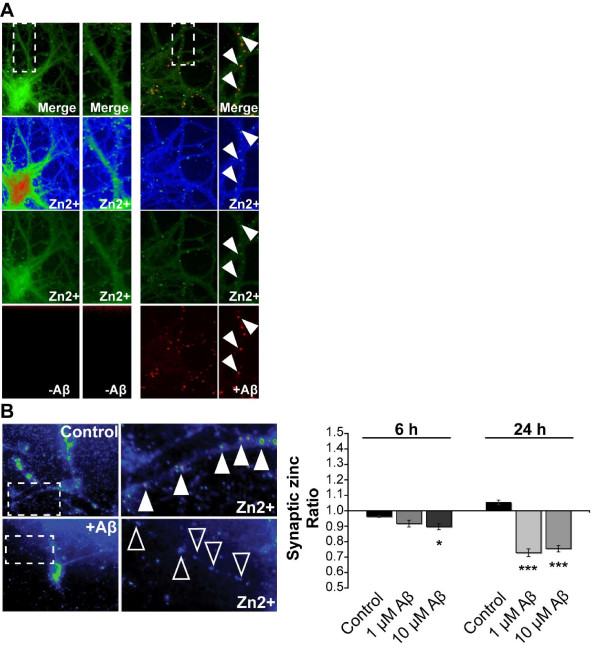
**Intracellular Zn^2+ ^levels are decreased in primary hippocampal culture neurons after treatment with soluble Aβ**. A) In hippocampal cell cultures (DIV15), a fraction of applied Aβ_1-40 _HiLyte Fluor™ 555 can be found intracellular, after removal of extracellular Aβ by washing steps, colocalizing with Zn^2+ ^stained by Zinpyr-1 (arrows). B) After treatment of hippocampal neurons (DIV15) with 1 μM and 10 μM Aβ_1-40_, a reduction in dendritic Zn^2+ ^signals (arrows) can be seen. The mean grey value of Zinquin signals inside dendrites was measured from five cells and the ratio between cells treated for 6 or 24 h and untreated cells is shown. A significant reduction can be seen after 6 h (10 μM) and 24 h (1 μM and 10 μM) treatment.

As the observed changes in synapse density and synaptic levels of ProSAP/Shank within 6-24 h after treatment with Aβ are relatively fast, we followed the possibility that intracellular Aβ contributes to a dysregulation of intracellular Zn^2+ ^levels in neurons. Indeed, application of fluorescently-tagged Aβ to hippocampal neurons in cell culture was followed by intracellular colocalization of Aβ and Zn^2+ ^(Figure 4A, arrows). Since these neurons were cultivated in medium without Zn^2+^-supplementation, Zn^2+ ^ions colocalizing with Aβ are most likely depleting other Zn^2+ ^stores. We therefore investigated postsynaptic Zn^2+ ^levels of hippocampal neurons after treatment with Aβ Zinquin labels postsynaptic Zn^2+ ^(Additional file 3A), which is in line with previous studies that revealed a striking colocalization of dendritic ProSAP2/Shank3 and Zinquin, colocalizing apposed to presynaptic boutons loaded with the styryl dye FM [17]. A significant reduction of Zn^2+ ^signals within dendrites (Figure 4B and Additional file 3B) was seen after Aβ treatment.

### Zn^2+ ^supplementation leads to a rescue of Aβ induced decrease in synapse density and ProSAP2/Shank3 levels at the synapse

**Figure 5:**
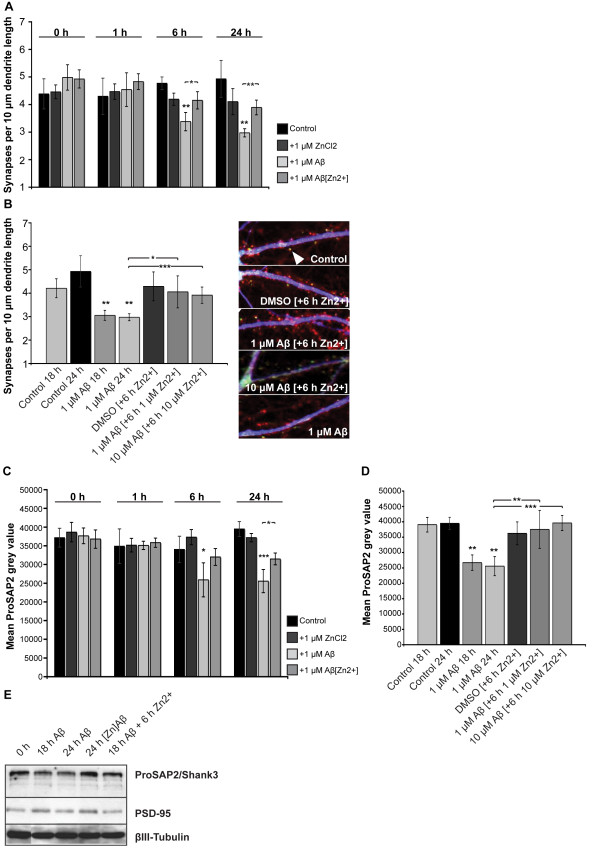
**Aβ binding of Zn^2+ ^regulates synapse loss and synaptic levels of ProSAP2/Shank3 in hippocampal cell culture**. A,B) Effect of Aβ and Zn^2+ ^on synapse density assessed by treating cultured hippocampal neurons (DIV15) with Aβ_1-40 _followed by equimolar ZnCl_2 _supplementation or Aβ_1-40 _preincubated with equimolar ZnCl_2_. Synapse density was determined by quantifying the number of ProSAP2/Shank3 and Bassoon colocalizing puncta per unit length of MAP2 positive primary and secondary dendrites (arrow, right panel B). A) Quantification of synapse density on neurons treated for 1, 6 and 24 h with DMSO (control/vehicle), 1 μM Zn^2+^, 1 μM Aβ_1-40 _and 1 μM Aβ_1-40 _preincubated for 1 h on ice with 1 μM ZnCl_2_. B) Treatment of hippocampal neurons for 18 and 24 h with DMSO or Aβ_1-40_, 18 h with DMSO and 6 h with 1 μM ZnCl_2 _and 18 h with 1 μM or 10 μM Aβ_1-40_, followed by 1 μM or 10 μM ZnCl_2 _supplementation for 6 h. Synapse density is significantly higher in cultures supplied with Aβ saturated with Zn^2+ ^than in those treated with 1 μM Aβ_1-40 _alone. C,D) Synaptic levels of ProSAP2/Shank3 in hippocampal cultures treated with Zn^2+ ^and/or Aβ C) Quantification of ProSAP2/Shank3 signal grey values colocalizing with Bassoon puncta along MAP2 positive primary and secondary dendrites of neurons treated for 1, 6 and 24 h with DMSO (control), 1 μM Zn^2+^, 1 μM Aβ_1-40 _and 1 μM Aβ_1-40 _preincubated for 1 h on ice with 1 μM ZnCl_2_. A significantly higher ProSAP2/Shank3 level compared to treatment with 1 μM Aβ_1-40 _was measured after 24 h in cultures supplied with Zn^2+^-saturated Aβ D) Neurons treated for 18 and 24 h with DMSO or Aβ_1-40_, 18 h with DMSO and 6 h with 1 μM ZnCl_2 _and 18 h with 1 μM or 10 μM Aβ_1-40_, followed by 1 μM or 10 μM ZnCl_2 _supplementation for 6 h. Supplementation of ZnCl_2 _for 6 h after 18 h treatment with Aβ_1-40 _leads to a rescue of ProSAP2/Shank3 levels at the synapse (p < 0.05*; < 0.01**; < 0.001***). E) Western blots of P2 membrane fractions from hippocampal neurons cultured for 15 DIV and then treated for 18 or 24 h with Aβ_1-40_, 24 h Aβ_1-40 _preincubated for 1 h on ice with ZnCl_2 _and 18 h Aβ_1-40 _followed by 6 h incubation with ZnCl_2_. Compared to untreated cells at time-point 0 h, a decrease in the amount of ProSAP2/Shank3 could be detected after 18 and 24 h of Aβ treatment. In contrast, treatment for 24 h with Zn^2+ ^saturated Aβ_1-40 _and 18 h Aβ_1-40 _followed by 6 h incubation with ZnCl_2 _leads to ProSAP2/Shank3 levels comparable to control conditions. Note PSD-95 and β-III Tubulin levels did not change under these conditions.

Based on the results obtained through the previous experiments, we investigated if Zn^2+ ^supplementation along with Aβ_1-40 _treatment or the saturation of Aβ_1-40 _with Zn^2+ ^before treatment led to a rescue of the observed changes in synapse density and ProSAP2/Shank3 protein levels at the PSD. Therefore, hippocampal neurons (DIV15) were treated with Aβ_1-40 _and supplemented with equimolar levels of ZnCl_2 _or with equimolar ZnCl_2 _preincubated with Aβ_1-40 _(Figure 5). Synapse density and protein levels of ProSAP2/Shank3 at the synapse were measured as described above (Figure 1). The results show that after treatment for 1, 6 and 24 h, neither control (DMSO-supplemented) nor 1 μM Zn^2+^-supplemented neurons display an increase or decrease in synapse density (Figure 5A, B). However, treatment with 1 μM Aβ_1-40 _resulted in a significant decrease of synapse density after 6 and 24 h (Figure 5A). In contrast, treatment of hippocampal neurons with 1 μM Aβ_1-40 _preincubated for 1 h on ice with 1 μM ZnCl_2 _led to a significantly higher synapse density compared to treatment with 1 μM Aβ_1-40 _after 6 and 24 h. Saturation of Aβ with Zn^2+ ^thus ameliorates the effects of Aβ on synapse density. To investigate, if supplementation of Zn^2+ ^after Aβ-induced decrease in synapse density can rescue the effects of Aβ we treated hippocampal neurons (DIV15) for 18 h with 1 μM or 10 μM Aβ_1-40_, followed by 1 μM or 10 μM ZnCl_2 _supplementation for 6 h, respectively (Figure 5B). ZnCl_2 _supplementation for 6 h alone did not induce changes in synapse density, whereas 1 μM Aβ_1-40 _treatment resulted in a significant reduction after 18 and 24 h. However, supplementation of ZnCl_2 _for 6 h after 18 h treatment with Aβ_1-40_, led to a significantly higher synapse density compared to cells treated with Aβ_1-40 _alone. In fact, the synapse density after ZnCl_2 _supplementation was not significantly different from control (DMSO-treated) cells (Figure 5B).

To assess if Zn^2+ ^supplementation or saturation of Aβ with Zn^2+ ^is able to rescue ProSAP2/Shank3 levels at the synapse, we measured ProSAP2/Shank3 signal grey values under the conditions described above and performed Western Blot analysis of protein levels. The results show that after treatment for 1, 6 and 24 h, neither control (DMSO-supplemented) nor 1 μM Zn^2+^-supplemented neurons display any changes in ProSAP2/Shank3 levels at the synapse (Figure 5C, D, for loading control of Figure 5D see Additional file 4A). Treatment with 1 μM Aβ_1-40 _resulted in a significant decrease of ProSAP2/Shank3 levels after 6 and 24 h (Figure 5C) compared to control (DMSO treated) cells. However, 24 h treatment of hippocampal neurons with 1 μM Aβ_1-40 _preincubated for 1 h on ice with 1 μM ZnCl_2 _led to significantly higher ProSAP2/Shank3 levels compared to treatment with 1 μM Aβ_1-40 _alone (Figure 5C, E). Thus, Zn^2+^**-**saturated Aβ causes less decrease of ProSAP2/Shank3 protein levels at the synapse. Similar to the experiments described above, we investigated if supplementation of Zn^2+ ^after Aβ protein induced decrease in ProSAP2/Shank3 levels is able to rescue the effects of Aβ. To that end, we treated hippocampal neurons (DIV15) for 18 h with 1 μM or 10 μM Aβ_1-40_, followed by 1 μM or 10 μM ZnCl_2 _supplementation for 6 h (Figure 5D, E). Zn^2+ ^supplementation for 6 h alone did not induce changes in ProSAP2/Shank3 levels, whereas 1 μM Aβ_1-40_, treatment resulted in a significant reduction. Supplementation of 1 μM or 10 μM ZnCl_2 _for 6 h after 18 h treatment with 1 μM or 10 μM Aβ_1-40 _respectively, led to a complete rescue of the decrease in ProSAP2/Shank3 levels (Figure 5D, E) and did not depend on enhanced protein synthesis (Additional file 4B). Interestingly, Zn^2+ ^supplementation was also able to rescue synaptic Shank1 and partially, although not significantly, synaptic Homer1 levels (Additional file 4C).

Previous studies have demonstrated that the Aβ-Zn^2+ ^binding site is localized within residues 6-28 and that histidines may serve as the principal sites of interaction [29], but interaction of Zn^2+ ^with the full-length Aβ_1-40 _and Aβ_1-42_, as well as the truncated Aβ_1-16 _and Aβ_1-28_, were reported [30]. Thus, we used Aβ_29-40 _as a control in our assays, because the C-terminus residues 29-40 do not seem to be affected by metal ion interactions [31]. The results showed that Aβ_29-40 _does not significantly reduce synapse density within 24 h of treatment (Additional file 4D) nor does it significantly affect synaptic ProSAP2/Shank3 levels (Additional file 4E). We also investigated the amount of cell death after Aβ_1-40_, Aβ_1-42 _and Aβ_29-40 _treatment. Our data show similar toxicity with respect to cell death in hippocampal cell cultures. For example, at 48 h, Aβ_1-40_, Aβ_1-42 _and Aβ_29-40_-treated cells showed signs of cell death (Additional file 4F), while no significant decrease in neuron number was seen after 24 h of treatment. This implies that cell death occurs independently from Zn^2+ ^dysregulation.

### Altered Zn^2+ ^and ProSAP/Shank levels in human AD and APP-PS1 mouse brain

**Figure 6:**
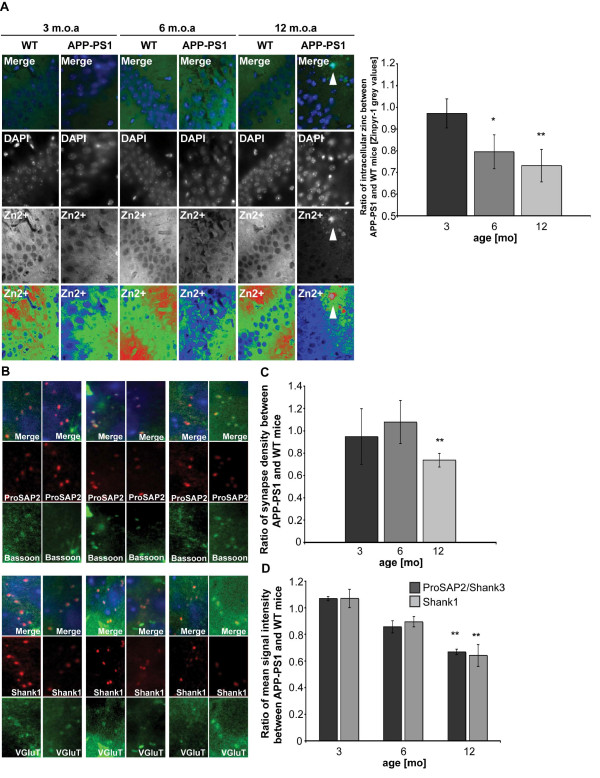
**Synapse density, synaptic ProSAP2/Shank3 and intracellular Zn^2+ ^levels are decreased in APP-PS1 mouse brain sections**. A) Cellular Zn^2+ ^levels are reduced in APP-PS1 hippocampus. Hippocampal sections from WT and APP-PS1 mice were double stained with DAPI to reveal cell nuclei and Zinpyr-1 to fluorescent-detect intracellular Zn^2+ ^(see Figure  S6). The ratio of mean Zinpyr-1 grey values between APP-PS1 and WT mouse sections at 3, 6 and 12 months of age is shown. The intracellular zinc concentration is significantly decreased in sections from 6 and 12 month-old mice (right panel). An enrichment of Zn^2+^-ions can be found colocalizing with extracellular Aβ plaques (arrow). B,C) The total number of synapses per optic field was measured and the ratio of mean number of synapses per optic field between APP-PS1 and WT mice is shown. A significant decrease is visible at 12 months. B,D) Sections of APP-PS1 and WT mice were stained with anti-Bassoon or anti-VGluT antibody as presynaptic marker and ProSAP2/Shank3 or Shank1 antibodies, respectively. The mean signal intensity of Alexa568 labeled ProSAP/Shank proteins opposed to a Bassoon or VGluT signal was measured and the ratio of mean grey values between APP-PS1 and WT mice is shown (merged images in B include DAPI staining (blue)).

**Figure 7:**
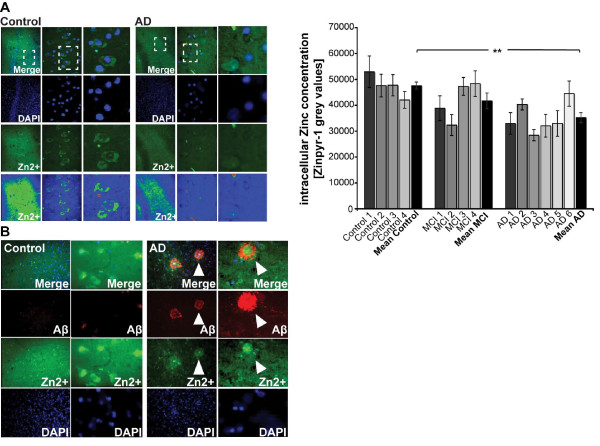
**Intracellular Zn^2+ ^levels are decreased in brain sections from AD cases**. A) Zinc ions were visualized by Zinpyr-1 and the fluorescence of intracellular Zn^2+ ^staining measured in AD patient and control sections (see Table 1). The mean Zinpyr-1 grey values of cells of 10 optical fields of view from AD and Control cases are shown (a, right panel). The intracellular Zn^2+ ^concentration is significantly decreased in sections of AD patients. B) Although the intracellular Zn^2+ ^concentration is decreased in AD, an enrichment of Zn^2+^-ions can be found colocalizing with extracellular Aβ plaques (arrow) in AD patient brain sections.

To assess whether an excess of Aβ leads to alterations of Zn^2+ ^levels *in vivo*, we visualized Zn^2+ ^using Zinpyr-1 in hippocampal brain sections of APP-PS1 mice (3, 6 and 12 months of age) and AD patients. The brightness of the intracellular Zinpyr-1 fluorescence that correlates with local Zn^2+ ^levels, was assessed (Figure 6A, 7A), as was the specificity of the signal by application of the Zn^2+ ^chelator TPEN (Additional file 5). After treatment with TPEN, the Zinpyr-1 signals in the CA3/dentate gyrus regions of the hippocampus were eliminated (Additional file 5). A comparison of Zn^2+ ^levels in the dentate gyrus and CA3 regions (Additional file 6) of wild type and APP-PS1 mice revealed a significantly lower Zn^2+ ^staining in APP-PS1 mice beginning with 6 m.o.a. (Figure 6A). Moreover, extracellular Zn^2+ ^ions were enriched in plaques formed by Aβ (Figure 6A, arrows). These data are consistent with higher Aβ levels causing a depletion of Zn^2+ ^in the hippocampus of older APP-PS1 mice. To assess whether disease progression in APP-PS1 mice is associated with a reduction in synapse density and/or synaptic ProSAP/Shank levels, we stained hippocampal sections from APP-PS1 mice with antibodies against ProSAP2/Shank3 or Shank1 as well as Bassoon and VGluT. In these experiments, we observed a significant reduction in synapse density in brain sections of APP-PS1 mice at 12 m.o.a. (Figure 6B, C). Similarly, synaptic levels of ProSAP2/Shank3 and Shank1 were significantly decreased at this age (Figure 6B, D).

To assess whether patients with Alzheimer's Disease (AD) exhibit similar reductions in Zn^2+ ^and ProSAP/Shank levels in the hippocampus, we analyzed brain sections from 14 control and AD patients (Table 1). Human sections were divided into three groups based on their Clinical Dementia Rating (CDR), Mini-Mental State Examination (MMSE), and Braak scores: a) "control" sections, b) sections of patients with mild cognitive impairment "MCI" and c) patients with terminal/severe Alzheimer's disease "AD". Similar to the experiments conducted in APP-PS1 mice, we assessed intracellular Zn^2+ ^concentration using Zinpyr-1 staining. The results show that sections from severely impaired AD patients ("AD") display significantly lower Zn^2+ ^staining (Figure 7A). Moreover, extracellular Zn^2+ ^ions were enriched at plaques formed by Aβ (Figure 7B, arrows). These observations are again consistent with the capacity of Aβ to bind, sequester and thus reduce intracellular Zn^2+ ^levels in the hippocampus of AD patients.

**Table 1:** Classification of human hippocampal brain sections

Case	Case Code	Age	Gender	Pmi [h]	CDR	MMSE	BraakNFT	BraakNP	Diagnosis
**Control 1**	99-111	75	M	6	0	29	1	1	C

**Control 2**	00-96	91	W	10	0	28	2	1	C

**Control 3**	02-55	82	M	4.5	0	28	2	1	C

**Control 4**	99-121	82	M	7	0.5	26	1	1	C

**MCI 1**	00-37	90	W	4	0.5	28	2	1	MCI

**MCI 2**	00-33	77	W	8	0.5	27	2	2	MCI

**MCI 3**	00-61	87	W	8	0.5	27	2	1	MCI

**MCI 4**	99-105	85	M	4.5	0.5	20	3	1	MCI

**AD 1**	99-68	88	M	8	2	13	3	3	AD

**AD 2**	00-58	85	M	6	2	20	4	2	AD

**AD 3**	99-96	90	M	2	2	10	6	3	AD

**AD 4**	98-15	85	W	11	3	11	6	4	AD

**AD 5**	99-67	95	W	3	3	0	5	4	AD

**AD 6**	99-98	102	W	11	3	0	6	4	AD

Hippocampal sections of human brains were used and classified as "Control", "MCI" and "AD" (m: man; w: woman; pmi: postmortem interval; CDR: Clinical Dementia Rating score; MMSE: Mini-Mental State Examination score; BraakNFT; neurofibrillary tangles Braak score; BraakNP: neuritic plaques Braak score; C: Control; MCI: mild cognitive impairment; AD: severe Alzheimer's disease).

**Figure 8:**
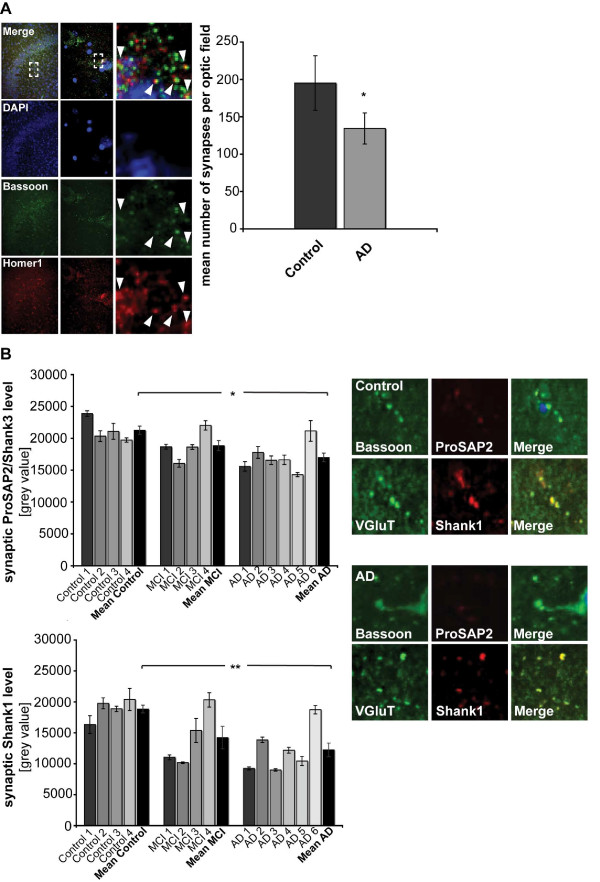
**Synaptic ProSAP/Shank protein levels are reduced during AD progression in hippocampal brain sections**. A) Immunofluorescent images of human hippocampal brain sections (CA3), stained with DAPI and antibodies against Bassoon and Homer1 (left panel) (low, medium and high magnification images are shown to reveal Bassoon/Homer1 co-clusters). The total number of synapses (colocalizing Bassoon/Homer1 puncta) was quantified to yield the mean number of synapses per optic field in control and patients with severe Alzheimer's disease (AD) (right panel). B) Loss of synaptic ProSAP2/Shank2 and Shank1 in AD patients was assessed by quantifying the intensity of ProSAP2/Shank3 and Shank1 puncta (mean grey values) colocalizing with Bassoon or VGluT (both presynaptic marker proteins) immunopositive puncta of hippocampal sections from control, "MCI" and "AD" patients.

To assess synapse density, we initially stained human hippocampal sections with antibodies against the presynaptic active zone protein Bassoon and the PSD protein Homer1. The number of Bassoon and Homer1 colocalizing puncta was then quantified per optic field (Figure 8A). This revealed a significant reduction in synapse number in brain sections of severe AD cases ("AD") compared to controls. To measure the change of ProSAP2/Shank3 and Shank1 at these synapses, the signal intensity of Alexa568-labeled ProSAP/Shank proteins opposed to a Bassoon or VGluT signal was measured (Figure 8B). Ten optical fields of 3 different sections per case were measured and the mean grey value per group calculated. The results showed a significant reduction of ProSAP2/Shank3 and Shank1 in the AD group compared to controls. Moreover, the clear trend towards this reduction could already be seen in "MCI" patient sections.

## Discussion

Soluble Aβ oligomers are thought to cause early synaptic damage and memory deficits in AD [32], although the mechanisms through which Aβ aggregates might lead to this phenotype are not yet fully understood. During the progression of AD, monomers of Aβ can aggregate to form amyloid fibrils. Five distinct fibrillar aggregates induced by Zn^2+ ^have been described [33], including protofibrils, Aβ-derived diffusible ligands (ADDL) and oligomeric species [34,35]. Oligomeric Aβ peptides have the ability to form dimers, trimers, tetramers and higher-order arrays that can form so-called annular structures. These are thought to influence the functionality of cytoskeleton-associated proteins, cause damage to synaptic spines and inhibit long-term potentiation in cultured neurons [33,36,37] and *in vivo *[38]. It was shown that physiological levels of Cu^2+ ^and Zn^2+ ^cause Aβ to aggregate [39] and that Zn^2+ ^ions are bound to Aβ via the histidine imidazole rings within senile plaque cores [40]. Recently, Adlard et al. proposed a mechanism whereby Aβ pathology causes cognitive impairment by trapping synaptic Zn^2+ ^rather than through direct toxicity [41]. Thus, the transsynaptic movement of Zn^2+ ^may be severely compromised in AD by being sequestered in Aβ. This trapping of Zn^2+ ^might mimic ZnT3 ablation [41] and indeed, mice with a disruption of the vesicular Zn^2+ ^transporter ZnT3, display complete absence of Zn^2+ ^from synaptic vesicles throughout the brain [42] as well as synaptic and memory deficits comparable to those seen in a cognitively impaired APP transgenic mouse model of AD [41].

Based on these findings, we propose a model, where Zn^2+^-ions might fail to reach their postsynaptic targets like ProSAP/Shank proteins due to sequestration by Aβ, leading to a dysregulation of the PSD scaffold and ultimately to a loss of synapses that can also be seen in ProSAP/Shank knockdown conditions [17]. This model is consistent with findings of Deshpande et al., who postulated that sequestration of Zn^2+ ^in oligomeric Aβ leads to reduced availability of Zn^2+ ^at the synapse, ultimately leading to cognitive deficits in AD [43]. To test this model, we investigated the influence of Aβ_1-40 _and Aβ_1-42 _on ProSAP/Shank family members in hippocampal neuron culture. In line with a number of recent publications showing the possibility that Aβ oligomers influence synaptic proteins and thus interfere with synaptic function [7,12,13,44,45], our study shows that the synaptic levels of ProSAP2/Shank3 and Shank1 decrease significantly following the addition of Aβ to primary neurons. Moreover, introduction of Aβ oligomers leads to a significant reduction (about 25%) in synapse density in hippocampal cultures, which is in agreement with previous studies reporting 11 to 77% declines in synaptophysin immunostaining in brain sections [46,47]. These results are also consistent with recent studies in cellular and rodent models, showing that small soluble oligomers are toxic because they directly damage synapses [32,48]. Furthermore, our experiments show that the loss of synapses is caused by a decrease in mature synapses. Thus, we conclude that the reduction in synapse density caused by Aβ is due to impaired activity dependent maturation and destabilization of mature synapses, but leaves the ability of an initial formation of synapses intact.

Additionally, treatment of hippocampal neurons with Aβ_1-40 _leads to a significant downregulation of ProSAP2/Shank3 at the synapse, to an impairment in synapse maturation and, in line with previous studies, to a downregulation of synaptic Shank1 levels [13]. The decrease in synaptic ProSAP2/Shank3 is also reflected by a decrease in protein levels in the P2 fraction as assessed by Western Blotting after 24 h treatment with Aβ_1-40_. Given the multiple interaction partners of ProSAP/Shank proteins at the synapse, it is likely that Aβ mediated changes in ProSAP/Shank complex formation cause synaptic dysfunction induced by reducing actin cytoskeletal assembly, spine motility as well as the maturation and plasticity of excitatory glutamatergic synapses.

We also show that the observed changes in ProSAP/Shank levels at the synapse are not due to altered gene expression, proteasomal degradation or protein synthesis and it appears that other posttranscriptional mechanisms control synaptic ProSAP/Shank levels. One interesting candidate is Zn^2+^, which is known to bind and regulate the synaptic localization of specific ProSAP/Shank family members, including ProSAP1/Shank2 and ProSAP2/Shank3 but not Shank1 [16-18]. We thus investigated whether an increased demand on extracellular Zn^2+^, e.g. by an increased level of Aβ, would reduce cellular levels of Zn^2+ ^and consecutively the synaptic levels of ProSAP/Shank family members. Using a cell-based assay, we directly demonstrated that the presence of extracellular Aβ interferes with the proper loading of ProSAP2/Shank3 with Zn^2+^. In contrast, saturation of Aβ with Zn^2+ ^before application does not change ProSAP2/Shank3 Zn^2+ ^loading.

In hippocampal cell culture, exogenously applied Aβ clusters with Zn^2+ ^intracellular and treatment of cultured neurons with Aβ reduces dendritic Zn^2+ ^levels. It was demonstrated previously that some intracellular Aβ is derived from extracellular Aβ pools and several distinct pathways of entry for extracellular Aβ have been proposed [49,50]. Although intracellular accumulation of Aβ is seen in multivesicular bodies and lysosomes, it can also be found within the cytosol [51]. Indeed, Kandimilla et al. have shown that Aβ is internalized by neurons primarily via passive diffusion [49]. That way, a fraction of intracellular accumulating Aβ might directly compete with Zn^2+ ^binding proteins such as ProSAP2/Shank3 for Zn^2+ ^ions in addition to the sequestration of extracellular Zn^2+ ^ions.

Based on these findings, we predicted that supplementation of hippocampal cultures with Zn^2+ ^during the treatment with Aβ or application of Zn^2+^-saturated Aβ would lead to a rescue of the observed loss-of-ProSAP2/Shank3 phenotype. Our results show that the Aβ-induced decrease in synapse density as well as lowered synaptic levels of ProSAP2/Shank3 can indeed be rescued by Zn^2+^-supplementation. Moreover, Zn^2+ ^saturated Aβ causes significantly less changes in synapse density and ProSAP2/Shank3 levels. Interestingly, also the decrease of Shank1 that shows a stronger requirement of NMDAR activity compared to ProSAP2/Shank3, can be rescued by Zn^2+^-supplementation. This indicates that Shank1 scaffold plasticity might depend on both, homeostatic changes via ProSAP2/Shank3 and the presence of Zn^2+ ^ions as well as on changes induced by synaptic activity, driven by the activation of downstream signaling pathways.

Our findings are further supported by *in situ *studies using APP-PS1 mice and AD patient brain sections. Here, we observed that Zn^2+ ^ions are enriched within amyloid plaques present in the hippocampus of older APP-PS1 mice and patients with severe AD. Intriguingly, intracellular Zn^2+ ^concentrations are ~20% lower in neurons from these sections compared to control sections. However, in addition to the sequestration of Zn^2+ ^by Aβ, other mechanisms may contribute to decreased intracellular Zn^2+ ^concentrations, for example Metallothioneins (MTs) or other Zn^2+^-binding proteins such as α2 macroglobulin (A2M) [52] may alter levels by regulating intracellular free Zn^2+^. MT upregulation, as reported for MT-I in AD mouse models [53], leads to inhibition of NO-mediated Zn^2+ ^release. Furthermore, pro-inflammatory cytokines cause a large induction of MTs [52]. Several Zn^2+ ^transporter proteins, including ZnT-1, ZnT-4 and ZnT-6, are altered in brain regions of subjects with early and late stages of AD [54]. Moreover, several members of the ZnT family (ZnT-1, 3, 4, 5, 6, 7) are expressed in amyloid plaques [55].

In addition to reduced intracellular Zn^2+ ^levels, we found a significant decrease in synapse density and synaptic ProSAP2/Shank3 and Shank1 protein levels. While chelation of Zn^2+ ^by extracellular Aβ appears a likely mechanism for influencing Zn^2+ ^levels in the brain, it should be noted that intracellular chelation of Zn^2+ ^might also contribute to its sequestration. Interestingly, it was recently found that serum Zn^2+ ^concentrations were significantly reduced from 12.3 μmol/l to 10.9 μmol/l in AD patients compared to control subjects [56]. Moreover, Zn^2+ ^supplementation greatly delays hippocampus-dependent memory deficits and strongly reduces both Aβ and tau pathology in the hippocampus of an AD mouse model [57].

However, distinct mechanisms might contribute to the observed decreases in PSD scaffold proteins in a brain region specific manner. In cortical cultures, the Aβ1-40-mediated reduction of PSD-95 protein levels is dependent on NMDAR activity and cyclin-dependent kinase 5, involving the proteasomal pathway [14]. However, the decreased levels of Homer1b and Shank1 were not influenced by proteasome activity. The decreased levels of synaptic Homer1b required *de novo *protein synthesis and involved the PI3-K pathway and calcineurin phosphatase (PP2B) activity, whereas declustering of Shank1 required NMDAR activity and activation of the ERK pathway [13]. In this study, the focus on the hippocampal region and the use of primary cultured neurons derived from hippocampus might explain the differences in regulatory pathways and kinetics mediating decreased levels of PSD scaffold proteins. This is underlined by our results, showing that a downregulation of ProSAP2/Shank3 and Shank1 in cortical neuronal cultures indeed occurs already after 1 h treatment with Aβ as reported previously [13]. Given that the hippocampus is the brain region with the highest Zn^2+ ^concentration, Zn^2+^-dependent regulatory mechanisms of PSD plasticity might be more pronounced in the hippocampus compared to other brain regions.

Although sporadic forms of AD are the most common, mutations in presenilin are associated with familial AD causing approximately 50% of these cases. In fact, it was recently reported that presenilin is important for cellular copper and zinc turnover, having the potential to affect Aβ aggregation indirectly through metal ion clearance [58]. Moreover, inflammatory processes that have been associated with AD [59] lead to a dysregulation of metallothioneins that might additionally sequester Zn^2+^. Thus, our experiments provide additional evidence for a common mechanism of the pathology of AD caused by the dysregulation of Zn^2+ ^levels within the brain.

## Conclusions

Based on our results and on recent studies [17], we conclude that Aβ complexes are able to bind extracellular and possibly also intracellular Zn^2+^, causing a dysregulation of Zn^2+^-dependent postsynaptic ProSAP/Shank scaffold proteins. Since ProSAP/Shank family members have specific roles in synapse formation and Shank1 is only targeted to a sufficiently preformed ProSAP1/Shank2-ProSAP2/Shank3 scaffold [17], the synaptic loss of ProSAP2/Shank3 could lead to instable synapse formation and/or maturation. This could further ultimately result in the untimely elimination of synapses [17,27] as evidenced by a reduction of Shank1 at the PSD in Aβ treated neurons and in patients with AD. In terms of cognitive performance, this is expected to affect the establishment of new memory and the retention of older memories during disease progression.

Although the idea, that sequestration of Zn^2+ ^by Aβ might cause the deficits seen in AD has been raised in the past, our data provide the first mechanistic insights, that could ty the dysregulation of a major postsynaptic scaffold molecule to the depletion of Zn^2+ ^by Aβ and consecutive synapse elimination.

## Methods

### Chemicals and reagents

Zinquin ethyl ester, ZnCl_2_, the Zn^2+ ^chelators CaEDTA and TPEN (*N,N,N',N'*-tetrakis(2-pyridylmethyl)-ethylenediamine) were purchased from Sigma-Aldrich. Zinpyr-1 was purchased from Mellitech. Primary antibodies were purchased from Covance (β-III Tubulin), Synaptic Systems (Homer1, PSD-95, VGluT), Novus Biologicals (Shank1 for IF), Stressgen (Bassoon), Sigma (PSD-95 for IF, Shank1 for WB) and Millipore (Aβ_1-40 _and Aβ_1-42_). ProSAP2/Shank3 antibodies have been described previously [17]. Secondary Alexa-coupled antibodies were from Invitrogen. Unless otherwise indicated, all other chemicals were obtained from Sigma.

### Hippocampal cultures from rat brain

The preparation of hippocampal cultures was performed essentially as described previously [60]. Cell culture experiments of hippocampal primary neurons from rat (embryonic day 18; E18) were performed as described previously [60]. After preparation, hippocampal neurons were seeded on poly-L-lysine (0.1 mg/ml; Sigma) glass coverslips. Cells were grown in Neurobasal medium (Invitrogen), complemented with B27 supplement (Invitrogen), 0.5 mM L-Glutamine (Invitrogen) and 100 U/ml penicillin/streptomycin (Invitrogen) and maintained at 37°C in 5% CO_2_. All animal experiments were performed in compliance with the guidelines for the welfare of experimental animals issued by the Federal Government of Germany and the National Institutes of Health. All of the experiments were conducted in strict compliance with APLAC approved animal protocols from Stanford University (protocol 14607) and by the local ethics committee at Ulm University (ID Number: O.103).

### Immunohistochemistry

For immunofluorescence, the primary cultures were fixed with 4% paraformaldehyde (PFA)/1.5% sucrose/1x PBS at 4°C for 20 min and processed for immunohistochemistry. After washing 3 × 5 min with 1x PBS at RT, blocking was performed with 0.5% cold fish gelatine (Sigma) and 0,1% ovalbumin (Sigma)/1x PBS for 30 min at RT and the cells were washed again 3 × 5 min with 1x PBS at RT, followed by the primary antibody at 4°C overnight. After a 3 × 5 min washing-step with 1x PBS, incubation with the second antibody coupled to Alexa488, Alexa568 or Alexa647 for 1 h followed. The cells were washed again in 1x PBS for 10 min and 5 min with ddH_2_O and mounted with Mowiol with or without DAPI (4',6-diamidino-2-phenylindole, for staining the nuclei) for fluorescence microscopy. Fluorescence images were obtained using an upright Axioscope microscope equipped with a Zeiss CCD camera (16 bits; 1280 × 1024 ppi) using the Axiovision software (Zeiss) or a spinning disk confocal microscope from Zeiss with MetaMorph (Universal Imaging) software.

### Human sections

Human brains from patients with different dementia severity were obtained from the autopsy service at the Department of Psychiatry from the University of Geneva, School of Medicine, Geneva, Switzerland. All procedures were reviewed and approved by the relevant Institutional Review Board and Ethics Committees. Details on the cases are provided in Table 1. Materials were fixed as full hemispheres in 4% paraformaldehyde for up to 6 weeks. Sections from hippocampal blocks were cut on a vibratome at a thickness of 50 μm and kept as free-floating series in PBS-azide at 4°C. For staining, sections were exposed to blocking solution, 10% BSA in 1x PBS for 1 h at room temperature and then incubated with the appropriate primary antibody in the blocking solution overnight at 4°C. The sections were washed with buffer and incubated with the secondary antibody (1:1000) in blocking solution for 1 h at room temperature. Afterwards, sections were mounted in VectaShield (Vector Laboratories) with DAPI for confocal fluorescence microscopy.

### Mouse sections

Animal studies were conducted in accordance with the National Institutes of Health guidelines for the use of experimental animals, and protocols were approved by the Institutional Animal Care and Use Committee. All mice were housed in an environment controlled for lighting (12-hour light/dark cycle), temperature, and humidity, with food and water available ad libidum. Male APP(swe)-PS1(dE9) mice, backcrossed for more than ten generations to a C57BL/6J background, were used for this study along with male non-transgenic littermates. At 3, 6, or 12 months of age, mice were deeply anesthetized and trans-cardially perfused with 0.9% saline. Brains were removed and fixed with 4% PFA in PBS for 24 h followed by immersion in 30% sucrose in PBS for more than two days. Coronal brain sections (40 μm) were prepared using a sliding microtome and used for immunostaining experiments as described above.

### Biochemical Analysis

To obtain P2/S2 fractions from hippocampal cultures, DIV15 cells exposed to different compounds of interest for the indicated times, were harvested and homogenized in homogenization buffer (320 mM sucrose, 5 mM HEPES, pH 7.4) containing protease inhibitor mixture (Roche). Cell debris and nuclei were removed by centrifugation at 1000 × g for 15 min. The supernatant was spun for 20 min at 12.0000 × g resulting in supernatant S2 (soluble fraction) and pellet P2 (membrane-associated fraction). Protein concentration was determined by amidoblack analysis and samples were further separated by SDS-PAGE, Coomassie-stained or blotted onto PVDF membranes using standard protocols. Immunoreactivity was visualized using HRP-conjugated secondary antibodies (DakoCytomation) and the SuperSignal detection system (Rockford).

### Treatment of hippocampal cells

Aβ_1-40_, Aβ_1-42 _(American Peptides), Aβ_29-40 _peptide (VWR International) and labeled Aβ_1-40 _(Aβ HiLyte Fluor™ 555-labeled, Anaspec) were prepared as described previously [13] and snap frozen at -20°C. As reported in several previous studies, the predominant aggregates in such preparations consist of low N-oligomers (mainly monomeric to tetrameric). Experiments were done with primary hippocampal neurons at DIV15-17 as indicated. Aliquots of Aβ were diluted in culture medium to a final concentration of 1 μM or 10 μM immediately before use.

To assess Aβ cell toxicity, hippocampal DIV15 neurons were treated with Aβ_1-40 _(Ctrl, +1 μM ZnCl_2 _or 1 μM CaEDTA), Aβ_1-42 _and Aβ_29-40 _for 48 h and fixed at time-points 0 h, 6 h, 24 h and 48 h. The number of cells per optic field was determined counting DAPI positive nuclei and the number of neurons assessed by MAP2 staining. The mean of five different fields of view was calculated for each condition and time-point.

### Synapse measurements

Pictures and were taken from neuronal synapses of hippocampal neurons with an upright Axioscope microscope equipped with a Zeiss CCD camera and a spinning disk confocal microscope from Zeiss. Quantification of fluorescence data was performed using MetaMorph (Universal Imaging), Image J 1.44e for Macintosh, Axiovision and Noam Ziv's Openview software.

Statistical analysis in this paper was performed using Microsoft Excel for Macintosh and tested for significance using *t *tests followed by ANOVA with an α level of significance set at 0.05 (< 0.05*; < 0.01**; < 0.001***). For evaluation, fluorescent puncta positive for a presynaptic marker (Bassoon, VGluT) and postsynaptic marker (ProSAP2/Shank3, Shank1, Homer1, PSD-95) along primary and secondary dendrites within the field of view were counted. Additionally, grey values and the signal area of post- and presynaptic proteins were measured and the results for the different conditions were evaluated in a blinded comparison. Pictures were all taken with the same acquisition time.

### Zinc staining

Zinypr-1 was stored as a 5 M stock solution in DMSO at -20°C. For cell culture neurons, growth medium was discarded and the cells were washed three times with HBBS. Hippocampal sections were incubated with a solution of 5 μM Zinquin ethyl ester or Zinpyr-1 in HBSS for 30 min. Zinpyr-1 (C_46_H_36_Cl_2_N_6_O_5_, MW: 823.22 g*mol^-1^) is a membrane-permeant fluorescent sensor for Zn^2+ ^with a high specificity and affinity for zinc (Kd = 0.7 ± 0.1 nM). Zinquin ethyl ester was stored as a 5 M stock solution in DMSO at -20°C. Hippocampal neurons were incubated with a solution of 25 μM Zinquin ethyl ester in HBSS for 20 min at 37°C [28].

### Cos7 cell assay

Cos7 cells were maintained in Dulbecco's modified Eagle's medium (DMEM) with high glucose (Invitrogen), supplemented with 10% (v/v) fetal calf serum, 2 mM L-glutamine and 5 μM ZnCl_2_. Cells were grown on commercially available chamber-slides (Nunc) treated with poly-L-lysine (0.1 mg/ml; Sigma). Transfection experiments with GFP-ProSAP2 (aa1-1806; full-size ProSAP2/Shank3) were performed using the transfection-agent Lipofectamine 2000 (Invitrogen) according to the manufacturer's recommendations. At 16 h post-transfection, zinc-staining using Zinquin ethyl ester was performed (t = 0 min) or cells were treated for 10 min with TPEN (t = 10 min) followed by either zinc-staining or application of 10 μM fluorescent Aβ_1-40 _(β HiLyte Fluor™ 555-labeled) for 20 min. After application of fluorescent Aβ_1-40_, an equimolar amount of ZnCl_2 _(10 μM) was supplemented for 20 min and subsequently, zinc-staining (t = 50 min) was performed; Alternatively, 10 μM fluorescent Aβ preincubated for 1 h with 10 μM ZnCl_2 _was applied for 20 min followed by supplementation of 10 μM ZnCl_2 _for 20 min and subsequent zinc staining. After this, cells were fixed with 4% PFA and mounted in VectaShield without DAPI.

### Quantitative Real-time PCR

Isolation of total RNA from primary neuronal cell cultures was performed using the RNeasy kit as described by the manufacturer. Isolated RNA was eluted in a total of 20 μl RNase-free water (supplied with the kit) and stored at -80°C.

For the reverse transcriptase-mediated PCR studies, first strand synthesis and real-time quantitative RT-PCR amplification were carried out in a one-step, single-tube format using the QuantiFast SYBR Green RT-PCR kit. Thermal cycling and fluorescent detection were performed using the Rotor-Gene-Q real-time PCR machine (model 2-Plex HRM) (Qiagen). The qRT-PCR was assayed in 0.1 ml strip tubes in a total volume of 20 μl reaction mixture containing 1 μl of undiluted total RNA, 2 μl of QuantiTect Primer Assay oligonucleotides, 10 μl of 2x QuantiFast SYBR Green RT-PCR Master Mix supplemented with ROX (5-carboxy-X-rhodamine) dye, 6.8 μl of RNase-free water (supplied with the kit) and 0.2 μl of QuantiFast RT Mix. RT. Amplification conditions were as follows: 10 min at 50°C and 5 min at 95°C, followed by 40 cycles of PCR for 10 s at 95°C for denaturation, 30 s at 60°C for annealing and elongation (one-step). During the extension real-time fluorescence measurements were recorded by the PCR machine, thus monitoring real-time PCR amplification by quantitative analysis of the fluorescence emission. The SYBR Green I reporter dye signal was measured against the internal passive reference dye (ROX) to normalize non-PCR-related fluctuations in fluorescence which occurs from reaction tube to reaction tube. Resulting data were analysed utilizing the hydroxymethylbilane synthase gene as an internal standard to normalize transcript levels. Cycle threshold (ct) values were calculated by the Rotor-Gene-Q Software (version 2.0.2). Cycle threshold values indicate the PCR cycle number at which the measured fluorescence of the indicator dye (SYBR Green I), accordant to the quantity of amplified PCR products, is increasing in a linear fashion above background. All qRT-PCR reactions were run in duplicates in three independent experiments and mean ct values for each reaction were taken into account for calculations of data analysis. To ascertain primer specificity a melting curve was obtained for the amplicon products to determine their melting temperatures. Melting curve was driven from 60°C to 95°C rising in 1°C steps while fluorescence was recorded continuously. For negative controls and to check for reagent contamination a complete reaction mixture was used in which the RNA sample was replaced by RNase-free water. Real-time quantitative PCR was carried out using oligonucleotides allowing to investigate expression of the following genes: Shank1 and ProSAP2/Shank3 (validated primer pairs, Quantitect primer assay, Qiagen). All consumables used for the extraction of total RNA and real-time PCR analysis were purchased from Qiagen.

## Competing interests

The authors declare that they have no competing interests.

## Authors' contributions

AMG and MJS designed the outline of this study and carried out all experiments in cell culture together with MS. MJS and PTU performed the biochemical and qRT-PCR analysis. AMG performed the staining of human and mouse brain sections. PRH provided human, KIA and NSW provided mouse-brain sections and participated with JDB and TMB in the design and coordination of the study. AMG and MJS performed all data analysis and jointly drafted the manuscript with MA, PRH, JDB, CCG and TMB. All authors read and approved the final version. AMG and MJS contributed equally to this study.

## Supplementary Material

Additional file 1 **Synapse number and protein composition of neurons treated with Aβ_1-42_**. Hippocampal neurons (DIV15) were treated with 1 μM Aβ_1-42 _(soluble oligomers) and fixed after 0, 6, and 24 h. Immunocytochemistry was performed using anti-ProSAP2/Shank3 with anti-Bassoon as presynaptic marker. Images were taken with the same acquisition time and the mean grey value and mean area of ProSAP/Shank signals opposite Bassoon signals was measured. A) The synapse density was calculated measuring the number of synapses per unit dendrite length of ten cells of three independent experiments for every time-point and condition. The ratio of the mean synapse density between treated and untreated neurons shows a significant decrease in synapse density starting at 6 h exposure to Aβ_1-42_. B) The ratio of mean grey values between treated and untreated neurons shows a significant downregulation of ProSAP2/Shank3 at the synapse after 24 h treatment with Aβ_1-42_. C) Cultured hippocampal neurons were immunostained with antibodies against Homer1 and PSD-95 and the ratio of mean grey values between treated and untreated neurons was measured after 0 h, 1 h and 24 h treatment with Aβ_1-40_. A significant decrease is seen after 24 h of treatment. D) The mean signal intensity of Homer1, ProSAP2/Shank3 or Shank1 signals opposite Bassoon puncta was measured at time-point 0 h, 1 h and 24 h after Aβ_1-40_-treatment of cortical neurons. The ratio of signal intensity between treated and untreated synapses is shown. A decrease of ProSAP2/Shank3 and Shank1 levels can be seen as early as 1 h after treatment. E) The mean area of ProSAP2/Shank3 or Shank1 signals opposite Bassoon puncta was measured after Aβ_1-40_-treatment. The change in the ratio of mean grey values per mean area between treated and untreated synapses (see Figure 1C) is based on a change in grey values, since the mean signal area is found to be the same for all time-points and conditions. The results show no significant changes between treated and untreated neurons. F) ProSAP2/Shank3 levels in immature vs. mature spines was measured using fluorescence grey values after 24 h Aβ_1-40 _treatment and compared to control conditions (24 h treatment with DMSO). Fluorescence grey values were normalized against presynaptic marker (Bassoon) grey values. Immature synapses show lower levels of ProSAP2/Shank3, increasing from filopodia-like to thin and mushroom/stubby spines. Treatment with Aβ_1-40 _significantly decreases the amount of ProSAP2/Shank3 in all spine types (significance indicated for comparison between "filopodia like" and "filopodia like after treatment", "thin" and "thin after treatment" etc.). Click here for file

Additional file 2 **Evaluation of PSD proteins after Aβ_1-40 _treatment**. A) Western blots of S2 soluble fractions from hippocampal neurons cultured for 15 DIV and then treated for 6 h and 24 h with Aβ_1-40 _(P2 fractions presented in Figure 2A). Compared to untreated cells at time-point 0, no decrease in the amount of ProSAP2/Shank3 and Shank1 could be detected after 6 h or 24 h of Aβ-treatment. Note, Homer1 and PSD-95 levels did not change. Lysates from 3 independent experiments were quantified via Western Blot analysis by measuring the integrated density. The values were normalized against β-III Tubulin and 0 h was set to 100%. B) The reduction of ProSAP2/Shank3 and Shank1 at the synapse is independent of both, proteasomal degradation and protein synthesis, since treatment with the proteasome inhibitor MG132 and protein synthesis inhibitor CHX did not prevent Aβ_1-40 _induced changes in synaptic signal intensities of ProSAP2/Shank3 and Shank1. MK801, a NMDAR antagonist showed a tendency to prevent Aβ_1-40 _induced changes in ProSAP2/Shank3 (although statistically not significant), but significantly decreased the amount of Aβ_1-40 _induced changes in Shank1 levels. The ratio between two sets of untreated control cells is shown and compared to the ratio between cells treated with Aβ and untreated cells as well as cells treated with MG132, CHX or MK801 in presence of Aβ and cells treated with MG132, CHX or MK801 alone. Click here for file

Additional file 3 **Zinquin signals of neurons treated with Aβ_1-40_**. A) Zinquin ethyl ester detects synaptic Zn^2+ ^signals. Co-labeling with FM dye reveals that the Zn^2+ ^staining is mostly opposed to FM, thus marking postsynaptic compartments. B) After treatment of hippocampal neurons with 1 μM and 10 μM Aβ_1-40_, a reduction in dendritic Zn^2+ ^signal area above a fixed fluorescent threshold can be seen. The mean area of Zinquin signals above a fluorescence limit was measured from five cells and the ratio between cells treated for 6 or 24 h and untreated cells is shown. A significant reduction can be seen after 6 h (10 μM) and 24 h (10 μM) treatment. Click here for file

Additional file 4 **Zinc supplementation experiments *in vitro***. A) Coomassie staining, showing that similar amounts of protein were loaded for the quantification of changes in synaptic protein levels by Western blot analysis presented in Figure 5E. B-D) Hippocampal neurons (DIV15) were treated with 1 μM Aβ_29-40 _and fixed after 0, 6, and 24 h. Immunocytochemistry was performed using anti-ProSAP2/Shank3 with anti-Bassoon as presynaptic marker. Images were taken with the same acquisition time and the mean grey value and mean area of ProSAP/Shank signals opposite Bassoon signals was measured. B) Effect of Aβ and Zn^2+ ^on ProSAP2/Shank3 levels are independent of protein synthesis. Cultured hippocampal neurons were immunostained with antibodies against ProSAP2/Shank3 and the ratio of mean grey values between treated and untreated neurons was measured after 24 h treatment with Aβ_1-40 _or Aβ_1-40 _with equimolar ZnCl_2 _supplementation (see Figure 5) with and without application of the protein synthesis inhibitor CHX (15 μM). The presence of CHX does not prevent rescue of ProSAP2/Shank3 levels by ZnCl_2 _supplementation. C) Cultured hippocampal neurons (DIV 15) were immunostained with antibodies against Shank1 and Homer1 and the ratio of mean grey values between treated and untreated neurons was measured after 24 h treatment with Aβ_1-40 _or Aβ_1-40 _with equimolar ZnCl_2 _supplementation (see Figure 5). ZnCl_2 _supplementation leads to a significant increase in Shank1 signal intensity at the synapse. Although Homer1 levels are also increased, there is still a significant difference to control cells and no statistically significant difference to Aβ treated cells. D) The synapse density was calculated measuring the number of synapses per unit dendrite length of ten cells of three independent experiments for every time-point and condition. The ratio of the mean synapse density between treated and untreated neurons shows no significant decrease in synapse density. E) The ratio of mean grey values between treated and untreated neurons shows no significant downregulation of ProSAP2/Shank3 at the synapse after 24 h treatment with Aβ_29-40_. F) Hippocampal neurons (DIV15) were treated with Aβ_1-40 _Aβ_1-42 _and Aβ_29-40 _and fixed after 0, 6, and 24 h. The number of cells per optic field was identified by DAPI staining of nuclei and labeling of neurons by MAP2. No significant reduction is seen after 24 h (magnification of typical field of view of Aβ_1-40 _treated neurons, right panel) (scale bar = 200 μm). G) However, after 48 h, Aβ_1-40 _Aβ_1-42 _and Aβ_29-40 _treated neurons show signs of cell death (arrows, magnification of typical field of view of Aβ_1-40 _treated neurons, right panel). Supplementation of cultures with 1 μM ZnCl_2 _or zinc depletion using the cell impermeable Zn^2+ ^chelator CaEDTA did not lead to any changes in the amount of cell death (scale bar = 200 μm). Click here for file

Additional file 5 **Zinpyr-1 staining of human brain sections**. Zn^2+ ^ions were visualized by Zinpyr-1 in human (and mouse, data not shown) brain sections and the fluorescence of intracellular Zn^2+ ^staining measured in control sections with and without application of TPEN prior to Zn^2+ ^staining. The fluorescence of the Zinpyr-1 dye is mostly absent in TPEN-treated section revealing the high specificity of the Zn^2+^-staining in brain sections. Click here for file

Additional file 6 **Zinpyr-1 staining of APP-PS1 mouse hippocampal brain sections**. Zn^2+ ^ions were visualized by Zinpyr-1 and the fluorescence of intracellular Zn^2+ ^staining measured in APP-PS1 mouse hippocampal brain sections from dentate gyrus (dg) and CA3 region in mice 3, 6 and 12 m.o.a (Inset on the upper right: p = pyramidal cells, g = granule cells). Click here for file
