# Supplementary figures and images for: Amyloid beta protein-induced zinc sequestration leads to synaptic loss via dysregulation of the ProSAP2/Shank3 scaffold
Source: Mol Neurodegener. 2011 Sep 22;6:65. doi: 10.1186/1750-1326-6-65 (PMC3189132; doi:10.1186/1750-1326-6-65)

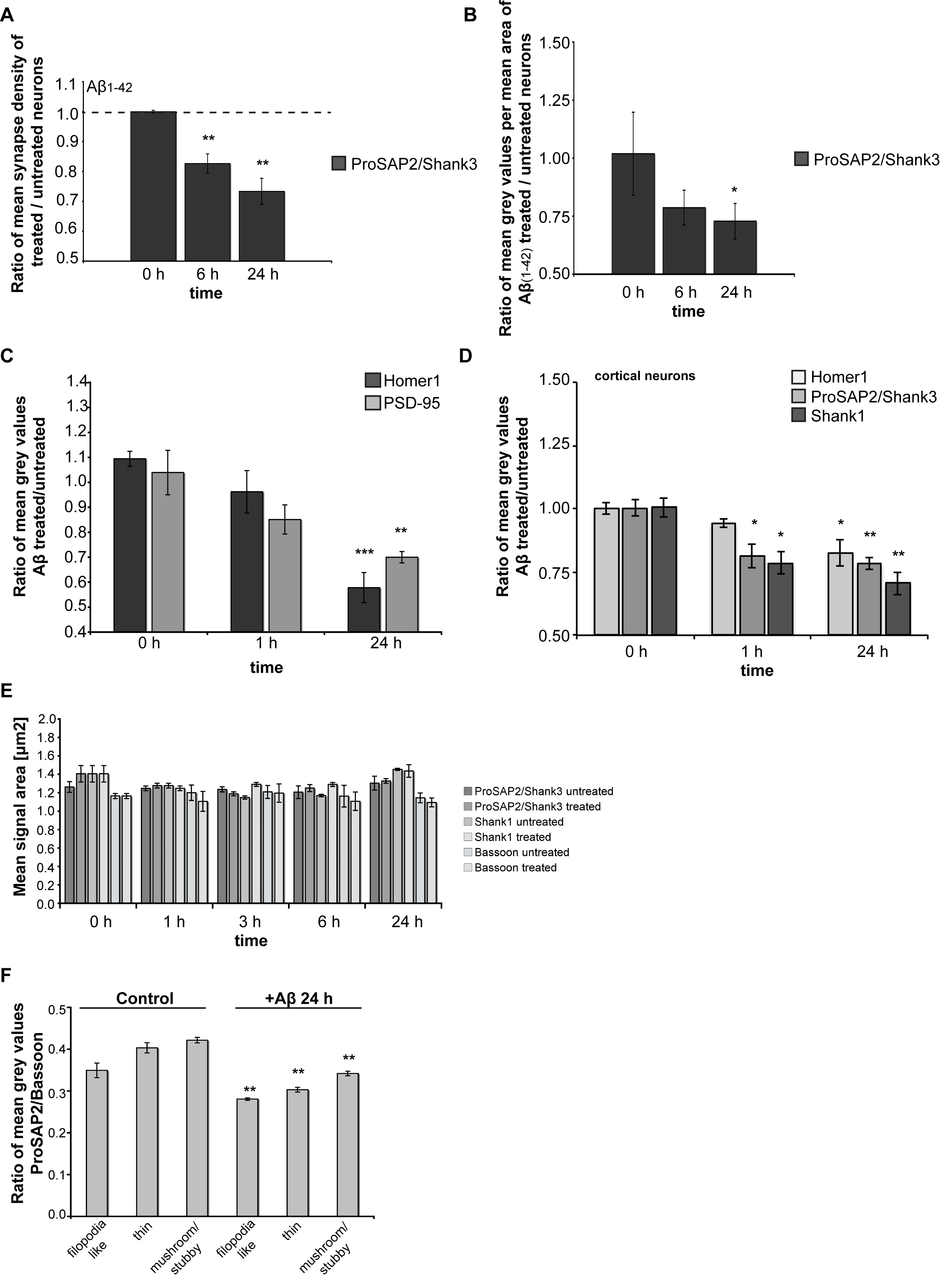

Supplement: Additional file 1 — Synapse number and protein composition of neurons treated with Aβ1-42. Hippocampal neurons (DIV15) were treated with 1 μM Aβ1-42 (soluble oligomers) and fixed after 0, 6, and 24 h. Immunocytochemistry was performed using anti-ProSAP2/Shank3 with anti-Bassoon as presynaptic marker. Images were taken with the same acquisition time and the mean grey value and mean area of ProSAP/Shank signals opposite Bassoon signals was measured. A) The synapse density was calculated measuring the number of synapses per unit dendrite length of ten cells of three independent experiments for every time-point and condition. The ratio of the mean synapse density between treated and untreated neurons shows a significant decrease in synapse density starting at 6 h exposure to Aβ1-42. B) The ratio of mean grey values between treated and untreated neurons shows a significant downregulation of ProSAP2/Shank3 at the synapse after 24 h treatment with Aβ1-42. C) Cultured hippocampal neurons were immunostained with antibodies against Homer1 and PSD-95 and the ratio of mean grey values between treated and untreated neurons was measured after 0 h, 1 h and 24 h treatment with Aβ1-40. A significant decrease is seen after 24 h of treatment. D) The mean signal intensity of Homer1, ProSAP2/Shank3 or Shank1 signals opposite Bassoon puncta was measured at time-point 0 h, 1 h and 24 h after Aβ1-40-treatment of cortical neurons. The ratio of signal intensity between treated and untreated synapses is shown. A decrease of ProSAP2/Shank3 and Shank1 levels can be seen as early as 1 h after treatment. E) The mean area of ProSAP2/Shank3 or Shank1 signals opposite Bassoon puncta was measured after Aβ1-40-treatment. The change in the ratio of mean grey values per mean area between treated and untreated synapses (see Figure 1C) is based on a change in grey values, since the mean signal area is found to be the same for all time-points and conditions. The results show no significant changes between treated and untreate [file 1750-1326-6-65-S1.TIFF]

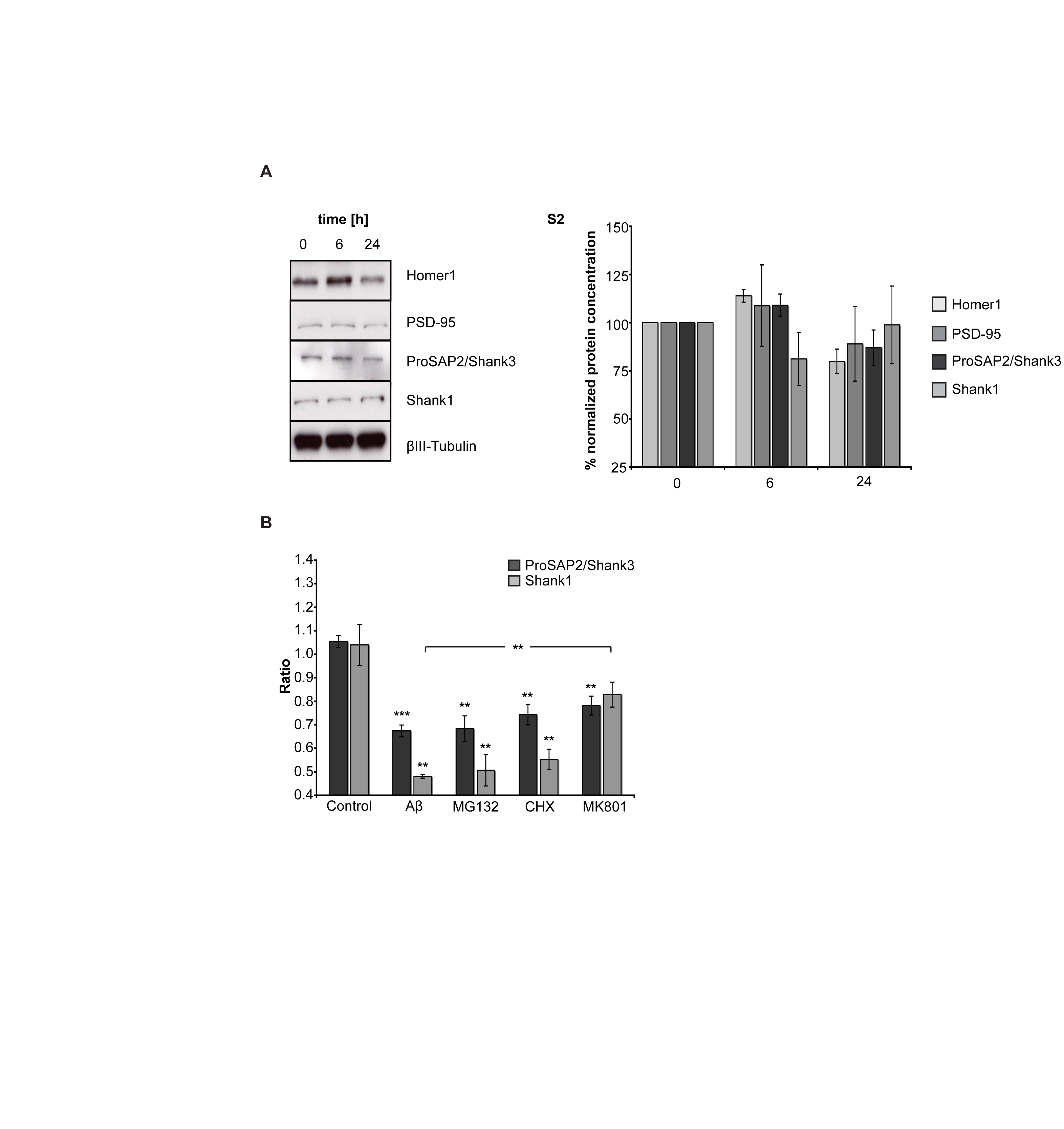

Supplement: Additional file 2 — Evaluation of PSD proteins after Aβ1-40 treatment. A) Western blots of S2 soluble fractions from hippocampal neurons cultured for 15 DIV and then treated for 6 h and 24 h with Aβ1-40 (P2 fractions presented in Figure 2A). Compared to untreated cells at time-point 0, no decrease in the amount of ProSAP2/Shank3 and Shank1 could be detected after 6 h or 24 h of Aβ-treatment. Note, Homer1 and PSD-95 levels did not change. Lysates from 3 independent experiments were quantified via Western Blot analysis by measuring the integrated density. The values were normalized against β-III Tubulin and 0 h was set to 100%. B) The reduction of ProSAP2/Shank3 and Shank1 at the synapse is independent of both, proteasomal degradation and protein synthesis, since treatment with the proteasome inhibitor MG132 and protein synthesis inhibitor CHX did not prevent Aβ1-40 induced changes in synaptic signal intensities of ProSAP2/Shank3 and Shank1. MK801, a NMDAR antagonist showed a tendency to prevent Aβ1-40 induced changes in ProSAP2/Shank3 (although statistically not significant), but significantly decreased the amount of Aβ1-40 induced changes in Shank1 levels. The ratio between two sets of untreated control cells is shown and compared to the ratio between cells treated with Aβ and untreated cells as well as cells treated with MG132, CHX or MK801 in presence of Aβ and cells treated with MG132, CHX or MK801 alone. [file 1750-1326-6-65-S2.TIFF]

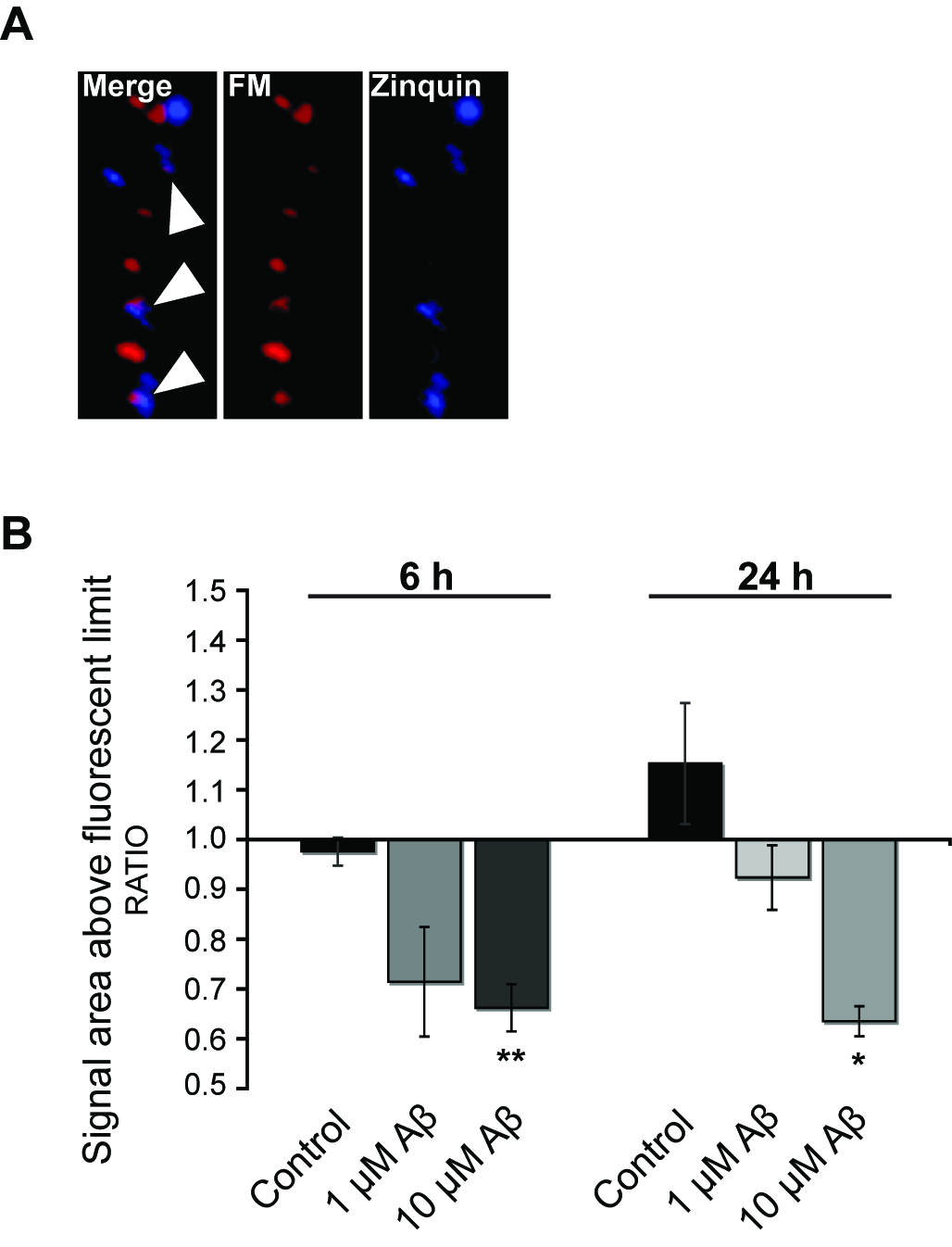

Supplement: Additional file 3 — Zinquin signals of neurons treated with Aβ1-40. A) Zinquin ethyl ester detects synaptic Zn2+ signals. Co-labeling with FM dye reveals that the Zn2+ staining is mostly opposed to FM, thus marking postsynaptic compartments. B) After treatment of hippocampal neurons with 1 μM and 10 μM Aβ1-40, a reduction in dendritic Zn2+ signal area above a fixed fluorescent threshold can be seen. The mean area of Zinquin signals above a fluorescence limit was measured from five cells and the ratio between cells treated for 6 or 24 h and untreated cells is shown. A significant reduction can be seen after 6 h (10 μM) and 24 h (10 μM) treatment. [file 1750-1326-6-65-S3.TIFF]

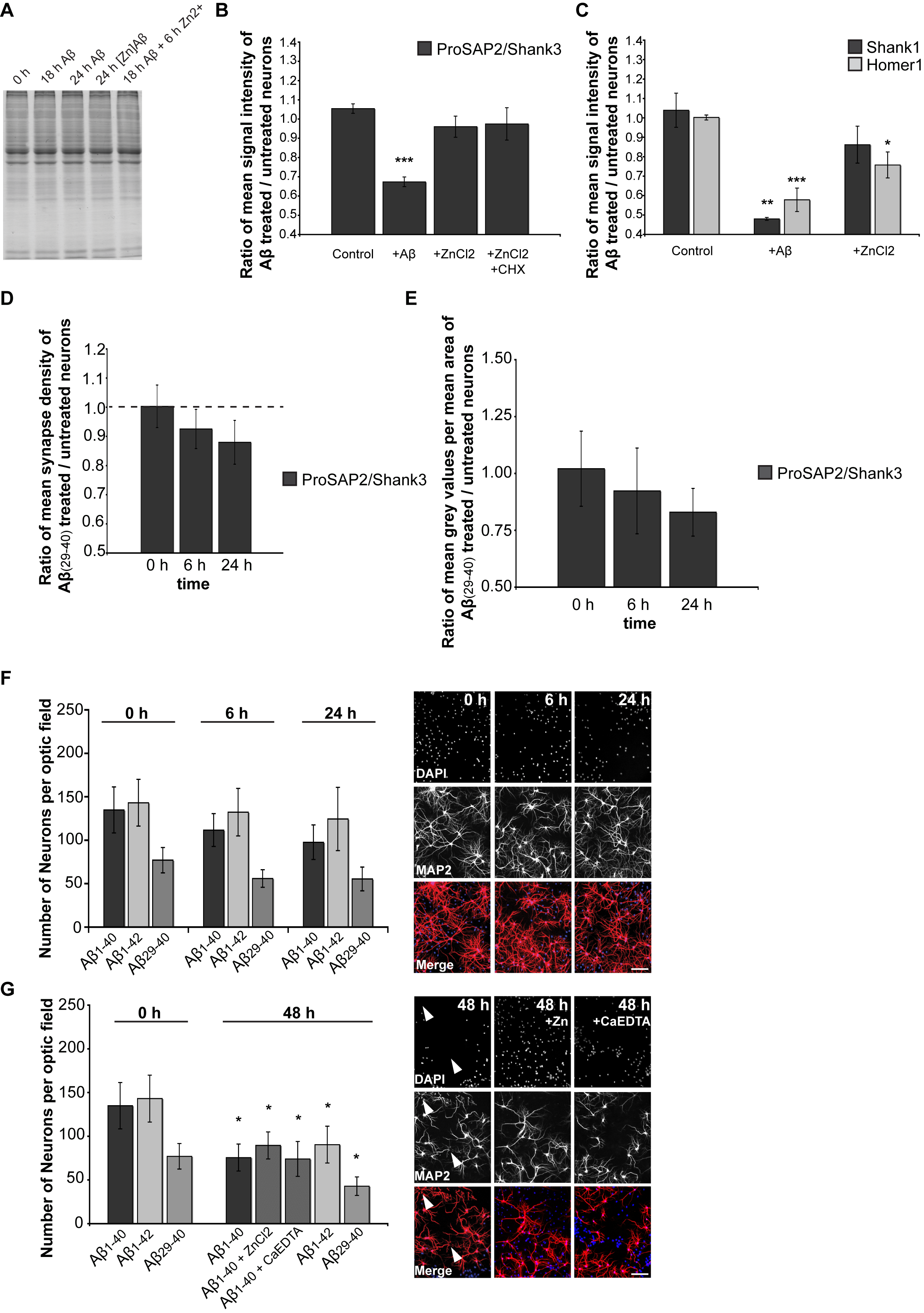

Supplement: Additional file 4 — Zinc supplementation experiments in vitro. A) Coomassie staining, showing that similar amounts of protein were loaded for the quantification of changes in synaptic protein levels by Western blot analysis presented in Figure 5E. B-D) Hippocampal neurons (DIV15) were treated with 1 μM Aβ29-40 and fixed after 0, 6, and 24 h. Immunocytochemistry was performed using anti-ProSAP2/Shank3 with anti-Bassoon as presynaptic marker. Images were taken with the same acquisition time and the mean grey value and mean area of ProSAP/Shank signals opposite Bassoon signals was measured. B) Effect of Aβ and Zn2+ on ProSAP2/Shank3 levels are independent of protein synthesis. Cultured hippocampal neurons were immunostained with antibodies against ProSAP2/Shank3 and the ratio of mean grey values between treated and untreated neurons was measured after 24 h treatment with Aβ1-40 or Aβ1-40 with equimolar ZnCl2 supplementation (see Figure 5) with and without application of the protein synthesis inhibitor CHX (15 μM). The presence of CHX does not prevent rescue of ProSAP2/Shank3 levels by ZnCl2 supplementation. C) Cultured hippocampal neurons (DIV 15) were immunostained with antibodies against Shank1 and Homer1 and the ratio of mean grey values between treated and untreated neurons was measured after 24 h treatment with Aβ1-40 or Aβ1-40 with equimolar ZnCl2 supplementation (see Figure 5). ZnCl2 supplementation leads to a significant increase in Shank1 signal intensity at the synapse. Although Homer1 levels are also increased, there is still a significant difference to control cells and no statistically significant difference to Aβ treated cells. D) The synapse density was calculated measuring the number of synapses per unit dendrite length of ten cells of three independent experiments for every time-point and condition. The ratio of the mean synapse density between treated and untreated neurons shows no significant decrease in synapse density. E) The ratio of mean grey values between treated [file 1750-1326-6-65-S4.TIFF]

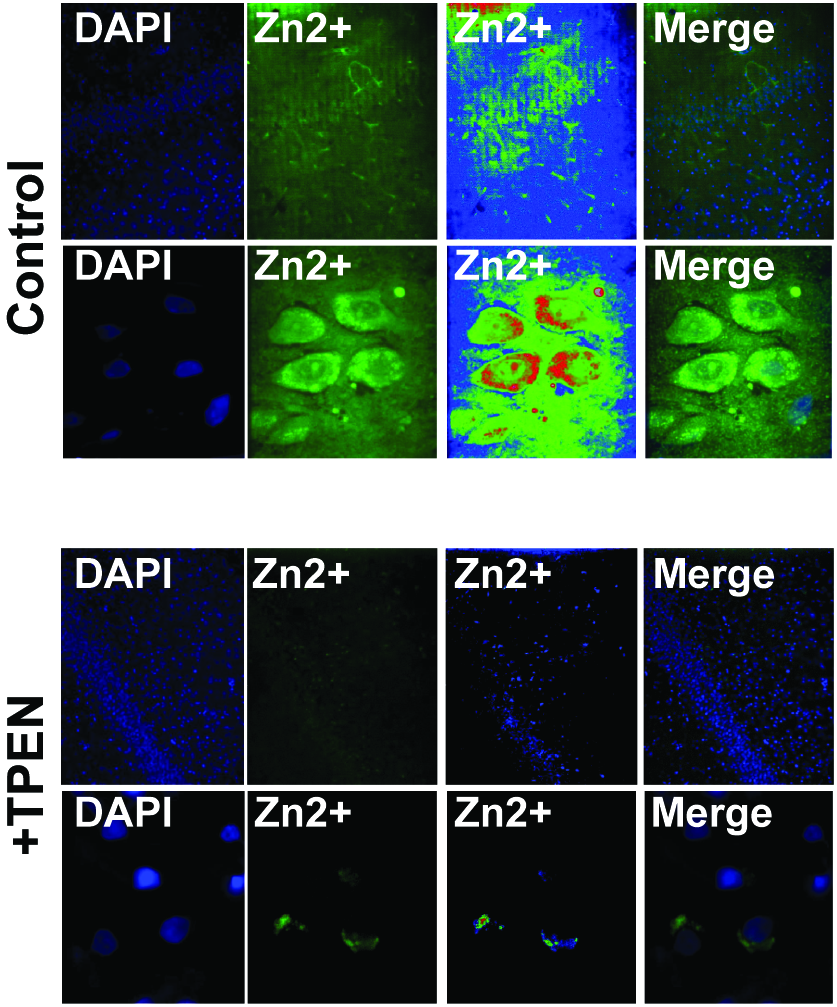

Supplement: Additional file 5 — Zinpyr-1 staining of human brain sections. Zn2+ ions were visualized by Zinpyr-1 in human (and mouse, data not shown) brain sections and the fluorescence of intracellular Zn2+ staining measured in control sections with and without application of TPEN prior to Zn2+ staining. The fluorescence of the Zinpyr-1 dye is mostly absent in TPEN-treated section revealing the high specificity of the Zn2+-staining in brain sections. [file 1750-1326-6-65-S5.TIFF]

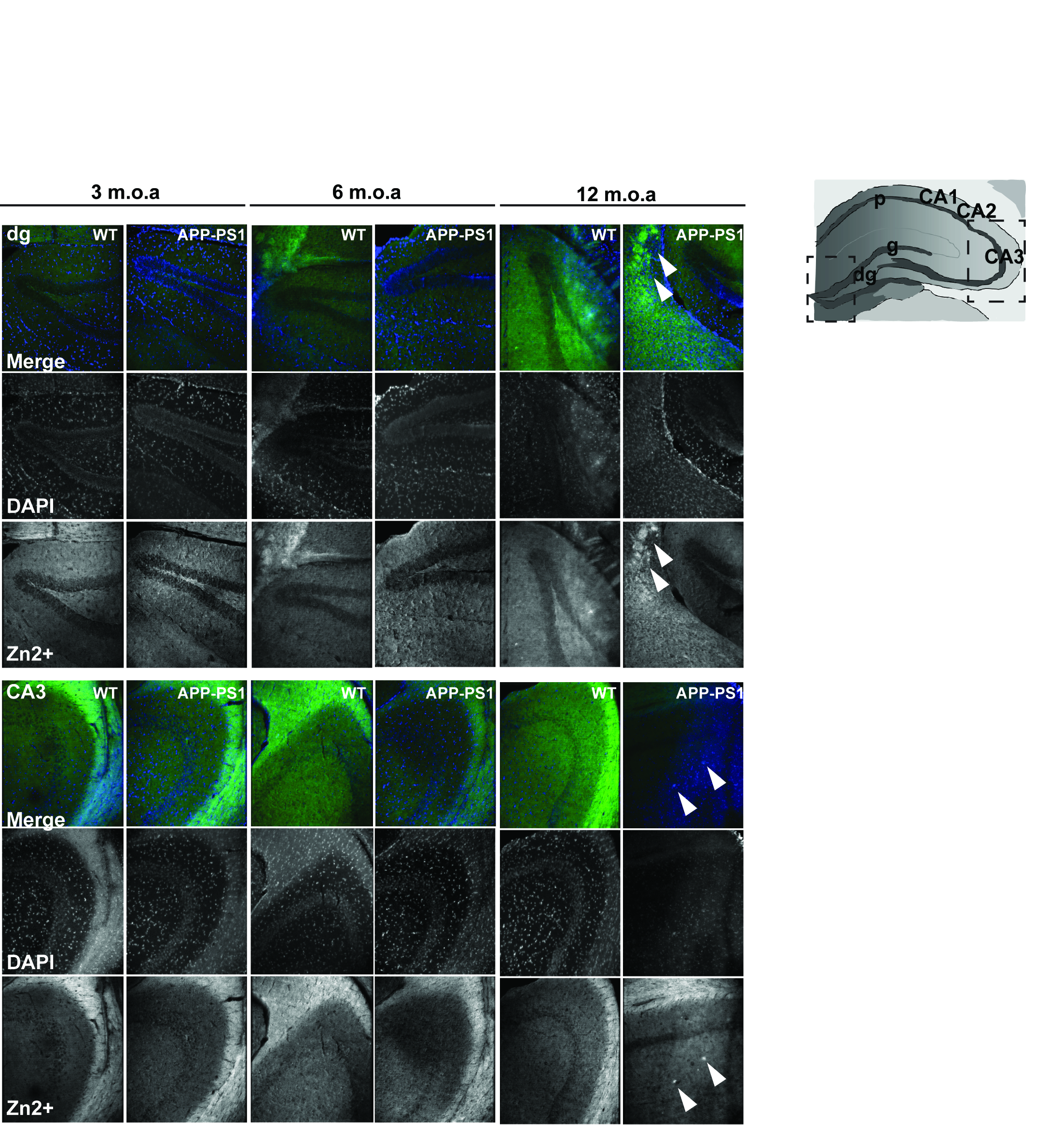

Supplement: Additional file 6 — Zinpyr-1 staining of APP-PS1 mouse hippocampal brain sections. Zn2+ ions were visualized by Zinpyr-1 and the fluorescence of intracellular Zn2+ staining measured in APP-PS1 mouse hippocampal brain sections from dentate gyrus (dg) and CA3 region in mice 3, 6 and 12 m.o.a (Inset on the upper right: p = pyramidal cells, g = granule cells). [file 1750-1326-6-65-S6.TIFF]
